# From Emergence to Endemicity: A Comprehensive Review of COVID-19

**DOI:** 10.7759/cureus.48046

**Published:** 2023-10-31

**Authors:** Roopa Naik, Sreekant Avula, Sujith K Palleti, Jyotsna Gummadi, Rajarajeshwari Ramachandran, Deepak Chandramohan, Gagandeep Dhillon, Angad S Gill, Kapil Paiwal, Bushra Shaik, Malavika Balachandran, Bhumika Patel, Simhachalam Gurugubelli, Abhishek Kumar Mariswamy Arun Kumar, Athmananda Nanjundappa, Mahita Bellamkonda, Kanika Rathi, Pavana Lalithya Sakhamuri, Mahmoud Nassar, Atul Bali

**Affiliations:** 1 Medicine, Geisinger Commonwealth School of Medicine, Scranton, USA; 2 Internal Medicine/Hospital Medicine, Geisinger Health System, Wilkes Barre, USA; 3 Diabetes, Endocrinology, and Metabolism, University of Minnesota, Minneapolis, USA; 4 Nephrology, Louisiana State University Health Sciences Center, Shreveport, USA; 5 Internal Medicine, MedStar Franklin Square Medical Center, Baltimore, USA; 6 Gastroenterology, The Brooklyn Hospital Center, Brooklyn, USA; 7 Nephrology, University of Alabama at Birmingham, Birmingham, USA; 8 Physician Executive MBA, University of Tennessee, Knoxville, USA; 9 Internal Medicine, University of Maryland Baltimore Washington Medical Center, Glen Burnie, USA; 10 Internal Medicine, Griffin Hospital, Derby, USA; 11 Oral & Maxillofacial Pathology, Daswani Dental College & Research Center, Kota, IND; 12 Internal Medicine, Onslow Memorial Hospital, Jacksonville, USA; 13 Internal Medicine, Wright State University, Dayton, USA; 14 Oral Medicine and Radiology, Howard University, Washington, D.C., USA; 15 Internal Medicine, Memorial Health System, Gulfport, USA; 16 Hospital Medicine, Kettering Health Network, Dayton, USA; 17 Hospital Medicine, University of Oklahoma Health Sciences Center, Oklahoma City, USA; 18 Internal Medicine, University of Florida, Gainesville, USA; 19 Rheumatology, Louisiana State University Health Sciences Center, New Orleans, USA; 20 Endocrinology, Diabetes, and Metabolism, Jacobs School of Medicine and Biomedical Sciences, Buffalo, USA; 21 Internal Medicine/Nephrology, Geisinger Medical Center, Danville, USA; 22 Internal Medicine/Nephrology, Geisinger Health System, Wilkes-Barre, USA

**Keywords:** vaccine, variants, virus, sars-cov-2, endemicity, emergence, covid-19

## Abstract

Severe acute respiratory syndrome coronavirus-2 (SARS-CoV-2), later renamed coronavirus disease 2019 (COVID-19), was first identified in Wuhan, China, in early December 2019. Initially, the China office of the World Health Organization was informed of numerous cases of pneumonia of unidentified etiology in Wuhan, Hubei Province at the end of 2019. This would subsequently result in a global pandemic with millions of confirmed cases of COVID-19 and millions of deaths reported to the WHO. We have analyzed most of the data published since the beginning of the pandemic to compile this comprehensive review of SARS-CoV-2. We looked at the core ideas, such as the etiology, epidemiology, pathogenesis, clinical symptoms, diagnostics, histopathologic findings, consequences, therapies, and vaccines. We have also included the long-term effects and myths associated with some therapeutics of COVID-19. This study presents a comprehensive assessment of the SARS-CoV-2 virology, vaccines, medicines, and significant variants identified during the course of the pandemic. Our review article is intended to provide medical practitioners with a better understanding of the fundamental sciences, clinical treatment, and prevention of COVID-19. As of May 2023, this paper contains the most recent data made accessible.

## Introduction and background

Coronavirus disease 2019 (COVID-19) was first identified in December 2019 and has since spread to nearly every corner of the globe [1]. The pathogenic COVID-19 infection is a novel coronavirus structurally related to the virus that causes severe acute respiratory syndrome (SARS). Because of its global spread, the World Health Organization (WHO) declared a pandemic on March 12, 2020 [1]. COVID-19 has had a devastating impact, claiming millions of lives. Even though vaccines were produced and delivered at an unprecedented rate, the virus has mutated and evolved, posing a threat to survival.

The effects were felt differently in different parts of the world, and even the most developed countries with robust healthcare systems and protocols were not immune [2]. To stem the spread of the disease, large-scale lockdowns were implemented in several parts of the world, along with preventive measures such as the widespread use of facial masks and the universal practice of hand hygiene. The economic burden imposed by COVID-19 was insurmountable in the form of healthcare costs, including vaccines, nationwide lockdowns, and industrial shutdowns, resulting in financial recession in most of the world [3]. COVID-19 has been found to affect almost all the organs in the body, causing a wide range of symptoms and posing a diagnostic challenge in several cases [4].

Because of extensive research and development worldwide, several vaccines and drugs have been developed to control infection spread and disease severity. Despite significant progress in mitigating the effects of COVID-19, as of March 31, 2023, the disease continues to spread globally, with the end game nowhere in sight [5]

## Review

Etiology

Coronaviruses are spherical, enveloped, positive-sense, single-stranded RNA viruses found in humans, other mammals, and birds [6]. The envelope contains glycoprotein spikes, giving the crown-like appearance under an electron microscope. Coronaviruses are divided into four main subgroups: alpha, beta, gamma, and delta. Alpha and beta coronaviruses likely originate from bats and rodents, whereas the gamma and delta variants likely come from the avian species [7].

**Figure 1:**
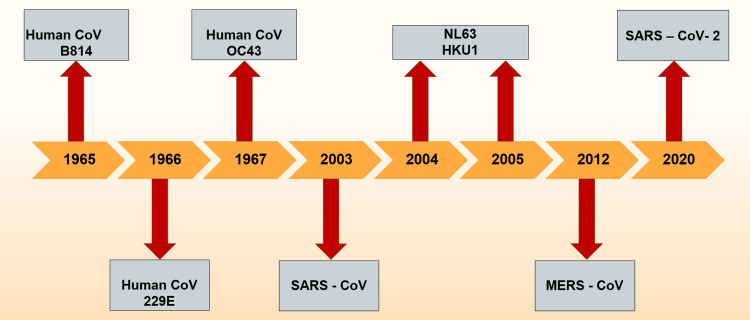
Timeline of detection of coronaviruses CoV: coronavirus; SARS: severe acute respiratory syndrome, MERS: Middle East respiratory syndrome

The first human coronavirus was identified in the mid-1960s. Since then, a total of seven coronaviruses known to infect humans have been identified (Figure 1); namely, 229E, alpha coronavirus (NL63), OC43, beta coronavirus (HKU1), severe acute respiratory syndrome coronavirus (SARS-CoV), Middle East respiratory syndrome coronavirus (MERS-CoV), and SARS-CoV-2; with the former four causing milder, self-limiting, upper respiratory symptoms and the latter three are known to be virulent and capable of widespread infections with clinical manifestations of varying severity [8].

SARS-CoV-2 is a novel coronavirus belonging to the beta subgroup. The virus resembles bat-derived coronaviruses, bat-SL-CoVZC45 and bat-SL-CoVZXC21 [9]. The virus is heat sensitive, although highly stable at 4℃ and with a wide range of pH at room temperature [3-10], but also susceptible to standard disinfection methods [10].

The origin of the virus remains unclear to date. Isolating closely resembling SARS-CoV-2 from horseshoe (*Rhinolophus*) bats of Yunnan caves in mainland China was a strong possibility; however, this theory was deemed unlikely given the geographical distance between the caves and Wuhan province, where the first case was identified [11]. Viruses appearing closely related to SARS-CoV-2 have also been identified in pangolins from China, Cambodia, Japan, and Thailand [12]. A possibility of spillover from bats to humans in Wuhan markets where animals, carriers of SARS-CoV-2, are sold alive for food was also postulated but later noted that the virus was not identified in these animals. Finally, there is widespread speculation that the virus escaped from the Wuhan Institute of Virology, which conducts research on SARS-related viruses. However, two different lineages of SARS-CoV-2 were simultaneously identified at different locations of Wuhan wildlife markets, leading to a likely natural origin for the virus with a yet-to-be-identified wild-caught or farmed animal [13].

Cross-species transmission is perhaps one of the most vital aspects of SARS-CoV-2 ecology as viral transmission from humans to animals has been documented in farmed minks, dogs, cats, and even wild animals including lions and tigers in zoos [14-16]. Upon animal infection, the human virus evolves and adapts to the new host. These recombinant human and animal coronaviruses may generate further novel viruses with a concern for spillover in humans, with partial or absent immunity, leading to a potential pandemic in the future [17].

COVID-19 variants and their characteristics

COVID-19 has had a devastating effect on the world's population, leading to millions of deaths. Since the beginning of the pandemic, SARS-CoV-2 has evolved, mutated, and produced variants with variable transmissibility and virulence, altering the performance of vaccines [18], diagnostic tools, therapeutic medicines, and other preventive measures.

SARS-CoV-2, an RNA virus, is prone to genetic evolution while adapting to their new human hosts with the development of mutations over time, resulting in the emergence of multiple variants [19]. The SARS-CoV-2 variants (Table 1) that emerged from the novel strain include Alpha (B.1.1.7), Beta (B.1.351), Gamma (P.1), Delta (B.1.617.2), and Omicron (B.1.1.529) [20].

**Table 1:** The list of variants of severe acute respiratory syndrome coronavirus 2 (SARS-CoV-2)

WHO Nomenclature	Lineage	Emergence
Alpha	B.1.1.7	Great Britain
Beta	B.1.351	South Africa
Delta	B.1.617.2	India
Gamma	P.1	Brazil
Epsilon	B.1.427	USA
Eta	B.1.525	USA
Iota	B.1.526	USA
Kappa	B.1.617.1	India
Mu	B.1.621	Columbia
Zeta	P.2	Brazil
Omicron	B.1.1.529	South Africa

**Figure 2:**
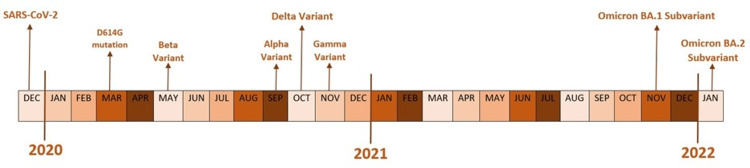
Timeline of severe acute respiratory syndrome coronavirus 2 (SARS-CoV-2) variants of concern

The timeline of the emergence of these variants is illustrated in Figure 2.

WHO designated SARS-CoV-2 variants with Greek alphabet letters according to their characteristics and classified them as given below [21].

Variants Being Monitored (VBM)

Variants having genetic changes suspected to affect virus characteristics with an indication that there may be a future risk associated with them. The variants in this category include Alpha (B.1.1.7 and Q lineages), Beta (B.1.351 and descendent lineages), Gamma (P.1 and descendent lineages), Delta (B.1.617.2 and AY lineages), Epsilon (B.1.427, B.1.429), Eta (B.1.525), Iota (B.1.526), Kappa (B.1.617.1), Unnamed (1.617.3), Omicron (B.1.1.529 and descendent lineages), Zeta (P.2), and Mu (B.1.621, B.1.621.1).

Variant of Concern (VOC)

Variants with increased transmissibility, virulence, or decreased effectiveness to available diagnostics, vaccines, and therapeutics. Omicron B.1.1.529, BA.1, BA.1.1, BA.2, BA.3, BA.4 and BA.5 lineages are listed as VOC.

Variants of Interest (VOI)

SARS-CoV-2 variants with genetic changes predicted or known to affect virus characteristics and have significant community transmissibility with emerging risk to global public health. There are currently no variants that are designated as VOI.

Variants of High Consequence (VOHC)

Variants with clear evidence of reduced effectiveness to medical countermeasures or preventative measures compared to the previously circulating variants. Currently, there are no variants designated as VOHC.

Alpha Variant

The Alpha variant was initially detected in the United Kingdom at the end of 2020, spreading rapidly in France, and soon became the dominant strain in March and June 2021 [22]. CDC classified it as a VOC once the variant was detected in the United States (US). It was reportedly 43-82% more transmissible than pre-existing variants of SARS-CoV-2 [23]. In mid-April in the US, Alpha comprised 66% of cases before delta emerged.

The most common symptoms reported were chills, headache, muscle aches, sore throat, fever, cough, and loss of appetite. However, loss of taste and smell were reported less commonly. The alpha variant was associated with increased disease severity, hospitalizations, and mortality compared to previous strains [24]. Vaccines effectively prevented severe disease and hospitalization in Alpha cases [25].

Beta Variant

The CDC classified Beta (B.1.351), alongside Alpha, as a VOC in December 2020 after it was discovered in South Africa in May 2020 [26]. Analysis of the spike substitutions revealed significant immune evasion, supported by reductions in neutralizing antibody titers in serum from individuals previously exposed to Beta-variant infections [27]. A significant infection surge in South Africa was noted due to the beta variant, leading to increased hospitalizations and mortality during the second wave. Moreover, it appears that vaccines are less successful at protecting against the Beta variant [28]. This variant is more contagious and produces more severe illness than the novel SARS-CoV-2 [29].

Infections from the Beta variant were associated with a 24% greater risk of severe illness, a 49% higher risk of critical illness, and a 57% higher risk of COVID-19-related death compared to infections with the Alpha variant. Acute-care admissions rose during the Beta wave and intensive care unit (ICU) hospitalizations and deaths quadrupled, with this variant having a disproportionately more significant impact on critical illnesses and COVID-19-related deaths [30].

Delta Variant

Delta variant was first identified in India in December 2020 [31], dramatically increasing COVID-19 and massive epidemics with record-breaking cases and deaths. Due to its strong transmissibility and ability to lead to an epidemic, it was classified as a VOC [20]. The Delta variant caused more than twice as many infections as the previous variants [32]. It was estimated to be 80-90% more transmissible than the Alpha variant. Researchers reported that the viral load was 1000 times higher in people infected with the Delta variant compared to people infected with prior variants of SARS-CoV-2, which could be related to its increased transmissibility [33].

The symptoms caused by the Delta variant did not differ from its predecessors. Delta caused more severe diseases than other variants. It has been observed that patients infected with this variant had higher hospitalization, ICU admissions, adverse events, and death compared to previous variants [34]. Several cases of mucormycosis, candidiasis, and aspergillosis were reported in India during the Delta variant outbreak.

Vaccination was highly effective against severe illnesses, hospitalizations, and deaths from the Delta variant [25]. Infections were detected even in fully vaccinated people. Since vaccinated patients with infections could spread the virus, the CDC recommended layered prevention strategies for vaccinated and unvaccinated people. In addition to being vaccinated, the public was advised to practice hand washing, wearing masks, and maintaining physical distance.

Delta AY.4.2

On June 11, 2021, Public Health England started covering the AY.1 sub-lineage of the Delta variant, which carries a second K417N mutation in the spike protein. "Delta Plus" is another name for this AY.1 variation. AY.4.2 had two mutations to its spike protein, AY145H, and A222V, but were not located where they would inhibit vaccine or treatment. It was considered 10-20% more transmissible than the Delta variant. No increased hospitalizations or deaths were noted. According to the CDC, vaccines are still quite successful in keeping people out of hospitals and preventing mortality against Delta and its sub-variants [35].

Omicron Variant

This is the most recent variant designated as a VOC by the WHO on November 26, 2021 [36]. Omicron has over 50 mutations in its genome, rendering it the most mutated SARS-CoV-2 variant. Omicron was discovered to have an effective (instantaneous) reproduction number of 3.19 (95%CI 2.82-3.61) times higher than the Delta variant. This variant was found soon after in Austria, Australia, Belgium, Canada, Czech Republic, Denmark, France, Germany, Italy, Netherlands, and the United Kingdom, with most cases being linked to travel [36].

The Omicron variant, a subtype of the Pango lineage B.1.1.529, has continuously undergone a sequence of unheard-of mutations and evolved to exhibit a wide range of characteristics [37]. Between 11 November and 14 November, 2021, variant B.1.1.529 was first discovered in Botswana, followed by South Africa [21]. It is a VOC because of its great transmissibility and low susceptibility to being neutralized by antibodies produced by prior viral exposure or vaccination. The Omicron variant's high infectivity and transmissibility properties are attributed to significant spike protein mutations. This variant's enhanced transmissibility is due to the substitutions N501Y and Q498R having a greater affinity for the angiotensin-converting enzyme 2 (ACE2) receptors, one of the receptor binding domain alterations [38].

Omicron is less severe than other variants, with a hospitalization risk that is 15-80% lower than the delta variant [39]. Omicron reproduces 70 times quicker than Delta in human bronchi while reproducing 10 times slower in human lung tissue, according to findings from ex vivo experiments. This explains why patients with infections from Omicron variants experience less severe disease [40]. It spawned a variety of sub-variants in 2022, including BA.5, BQ.1, and BQ.1.1 [41]. By January 2023, most infections in the US were being caused by a new Omicron subvariant known as XBB.1.5. Up to February 2023, Omicron and its sub-variants have been the most common SARS CoV-2 strains in the US [41].

The WHO and other organizations have put forth strategies to stop the establishment and spread of these novel variations. These include genomic surveillance at the local level, better access to anti-SARS-CoV-2 vaccines, widespread availability of face masks, quicker identification of infected patients, and implementation of appropriate containment strategies. To stop the creation of new SARS-CoV-2 variations, it is crucial to put these techniques into practice.

A brief history of the origins

The SARS-CoV-2 pandemic is estimated to have started on December 1, 2019, but its exact origins are still unknown and subject to intense scientific and political dispute [42-44]. Although the virus was believed to have most likely spread from a marine food market in Wuhan, Hubei Province, China, there is currently no convincing evidence to support this, and controversies still exist [45]. An outbreak of severe pneumonia with an unknown organism was reported to the WHO on December 31, 2019, in Wuhan, Hubei Province, China, a city with a population of approximately 11 million [46]. About 66% of the initial 41 patients admitted to the hospital with an unidentified pneumonia infection by January 2, 2020, had direct exposure to the Huanan Wholesale Seafood Market (hereafter, "Huanan market") [47].

An international team of scientists assembled by the WHO visited Wuhan, China, in January 2021, the location of the first discovery of the virus that causes COVID-19. As part of phase one research, the team evaluated data on when and how the virus may have evolved in collaboration with Chinese researchers [48]. The combined international team evaluated the possible routes for introducing the virus and issued several suggestions for each route: (i) Introduction by a direct zoonotic spillover was considered a possible route, (ii) Introduction through an intermediate host was regarded as a probable-to-very likely pathway, (iii) Introduction through products in cold chain or cold storage was thought to be a plausible method, and (iv) Introduction through a laboratory incident was thought to be a very unlikely method [49].

Introduction via Intermediate Host Followed by Zoonotic Transmission

In this hypothesis, SARS-CoV-2 spreads within the intermediate host (also known as the spillover host) after being transmitted from an animal reservoir to an animal host and then spreads to humans. Without (top row of animals) or with virus adaptation (bottom row of animals), the virus can pass through an intermediate host [45].

Direct Zoonotic Spread

In this hypothesis, the transmission of SARS-CoV-2 (or a closely related progenitor virus) from an animal reservoir host to a human in this instance was followed by direct person-to-person transmission with (top row of human icons) or without (bottom row) the necessity for the virus to adapt to humans [49].

Introduction Through the Cold/Food Chain

Food-chain transmission might represent spillage through an intermediary host or direct zoonotic transmission. In the meantime, cold chain products might be a means of human transmission. This would include both introductions and instances of food contamination. It is crucial to distinguish between cold chain product contamination causing additional outbreaks in 2020 and the possibility that cold chain served as the entry point for the pandemic's origin in 2019 [49].

Introduction Through a Laboratory Incident

In this hypothesis, a laboratory incident introduced SARS-CoV-2, reflecting an accidental infection from staff during the laboratory activity involving the relevant viruses. This did not consider the theories of deliberate release or intentional bioengineering of SARS-CoV-2 for release, the latter debunked by other researchers after genome analysis [49].

The WHO halted its phase-two ambitions after the well-publicized trip in 2021. According to the news article published in Nature on February 14, 2023, WHO discreetly abandoned the second stage of its highly anticipated scientific investigation into the origins of the COVID-19 pandemic, citing persistent difficulties with attempting to carry out crucial investigations in China [49]. According to Maria Van Kerkhove, an epidemiologist at the WHO in Geneva, Switzerland, phased work was planned by the WHO, but "that plan has changed," she claimed [49]. Chinese officials disapproved of the WHO's objectives, particularly the proposal to investigate lab breaches. According to Zhao Lijian, the spokesperson for China's foreign ministry, not all member nations concurred with the WHO plan, and the second phase shouldn't focus on possibilities that the mission report had previously considered highly implausible [48].

It has been determined that the epidemic was most likely caused by a laboratory leak in Wuhan, China, although with "low confidence" [50]. According to the federal government, low confidence refers to "limited, questionable, fragmented, or that strong analytical conclusion cannot be formed from the evidence." Moreover, there is currently no consensus within the American intelligence community regarding the origin of SARS-CoV-2 [44].

Economic burden of COVID-19

The COVID-19 pandemic created a health crisis and an economic burden worldwide. While quantifying the total economic impact, direct medical costs, loss of productivity, and costs due to non-pharmaceutical interventions like lockdown or mitigation strategies must be included [51]. At the beginning of the pandemic, economic data was scarce. Still, due to the extent of economic impact, several studies have been published from different regions, providing some insights into costs [52-55].

Lockdown Cost

During the initial phase of the pandemic, various countries implemented mitigation strategies of varying intensity and timing. A cost-benefit analysis of the lockdown in the United Kingdom saw a 68 billion pound loss even with a best-case scenario and, in the worst scenario, a 547 billion loss based on different gross domestic product loss assumptions and quality-adjusted life year (QALY) loss assumptions [56].

Pre-Vaccination Cost

The pandemic has had a major impact on contact-intensive sectors like health and social care, hospitality, recreation, retail and wholesale, and transportation. Rand Corporation ran several scenarios to find out how vaccination availability will affect the gross domestic product (GDP) of various countries' economies. In one scenario, when the world economy allowed activity without effective vaccination, it was projected to cause contact-intensive service sectors to lose about $3.4 trillion globally in GDP terms annually. This corresponded to about 3.7% of the global GDP and 2.2% of the US GDP [56].

Direct Healthcare Cost

US: In the early days of the pandemic, a study used the Monte Carlo simulation model to estimate potential healthcare costs in the US population. An estimated median direct medical cost was $3,045 during the course of infection, and total direct medical costs over the course of the pandemic ranged from $163.4 billion if 20% of the US population got infected to $654.0 billion if 80% of the US population was infected [57].

Worldwide: The cost of COVID-19 treatment had a disastrous economic outcome for many families in low and medium-economic countries. A study from Greece estimated that the cost per non-ICU patient and ICU patient were €8,852 and €24,167, respectively [53]. Cost analysis of 3254 patients with suspected or confirmed COVID-19 hospitalized in a large public hospital in São Paulo, Brazil, between March 30 and June 30, 2020, showed an average cost of $12,637.42 [54]. A study reviewed 745 COVID-19 patients treated in Iran, with an estimated mean total cost of $8813.15, accounting for 60% of the per capita [58]. Another study evaluated the medical records of 400 patients admitted to an Iranian hospital for COVID-19 treatment, with an estimated mean cost of treatment at $1,434 [55]. Another study from Turkey analyzed invoices of non-ICU and ICU patients and found the mean cost for non-ICU patients was $881.75 ± 667.31 (range: $45.07 - $7584.81), whereas the mean cost for ICU patients was $2924 ± 2347.14 (range: $223.01- $9681.88) [51,59].

Testing

Cost analysis of 598,502 COVID-19 samples collected for tracing and diagnosis in Addis Ababa, Ethiopia, showed the cost of COVID-19 sample collection, per COVID-19 positive individual was $11.63, and the cost of identifying COVID-19 positive cases by contact tracing was $54 [60].

Indirect Cost (Loss of Revenue and Productivity)

During the initial stages of the pandemic, due to the lack of adequate testing and PPE, elective procedures were canceled, leading to a loss of revenue for the healthcare system. A retrospective study compared the total revenue generated between the pre-COVID-19 era and the COVID-19 era and found a 55.7% decline in revenue generated by the orthopedic division of a single large tertiary hospital in Pakistan [61].

Vaccination Cost (Development, Production, and Administration)

Estimating the total cost of COVID-19 vaccination is challenging due to the various types of vaccinations and different deployment strategies used by various countries. As a part of Operation Warp Speed (OWS), the US government invested around $18 billion for the development and clinical trials for a total of six vaccine candidates from Moderna, AstraZeneca/Oxford, Johnson and Johnson, and Sanofi/GSK. BioNTech has received $445 million in funds from the German government to develop COVID‐19 vaccine development. The US purchased 200 million doses of the Moderna vaccine at $30 per person and 400 million doses of the Pfizer‐BioNTech vaccine at $39 per person [62].

A study by Ghana's Ministry of Health’s technical working group for health technology assessment using WHO-United Nations Children's Fund (UNICEF) COVID-19 vaccine introduction and deployment casting (CVIC) estimated cost of $20.9-$26.2 per person to complete the primary vaccination schedule, which included the cost of the vaccine [63].

Age, gender, and racial disparities

Age is a significant predictor of COVID-19 severity and mortality [64,65]. According to the CDC data, age is the strongest risk factor for severe COVID-19 outcomes, with the risk of death increasing exponentially with age [66]. Compared with individuals aged 18-29 years, the risk of death is 25 times higher in those aged 50-64 years, 60 times higher in those aged 65-74 years, 140 times higher in those aged 75-84 years, and 340 times higher in those aged 85 years or older [67].

Recent studies indicate that individuals who identify as male are at a higher risk of mortality from COVID-19, possibly due to androgen-mediated regulation of the transmembrane protease serine 2 (TMPRSS2) and ACE-2, which could facilitate the entry and replication of SARS-CoV2 [64,68,69]. Other potential explanations for this gender disparity include differences in immune and inflammatory reactions and clotting and bleeding tendencies [69].

COVID-19 infections and mortality risk were found to be higher among Black and minority ethnic groups compared to White individuals. Despite identifying differences in socioeconomic determinants and comorbidities such as cardiovascular disease and diabetes as contributing factors, they do not fully account for the observed disparities [64,70,71]. Further investigation is needed to elucidate the underlying factors.

COVID-19 pathophysiology

Coronaviruses are spherical, enveloped, positive-sense, single-stranded RNA viruses found in humans, other mammals, and birds [6]. Six species are known to affect humans, of which four viruses (229E, OC43, NL63, and HKU1) predominantly lead to common cold symptoms, mostly in immunocompetent individuals [72]. The other two strains are SARS-CoV-1, responsible for the 2002-2003 SARS-CoV pandemic, and the MERS-CoV found first in the Arabian peninsula in 2012 [73]. The third strain is the SARS-CoV-2, leading to the global outbreak of COVID-19.

**Figure 3:**
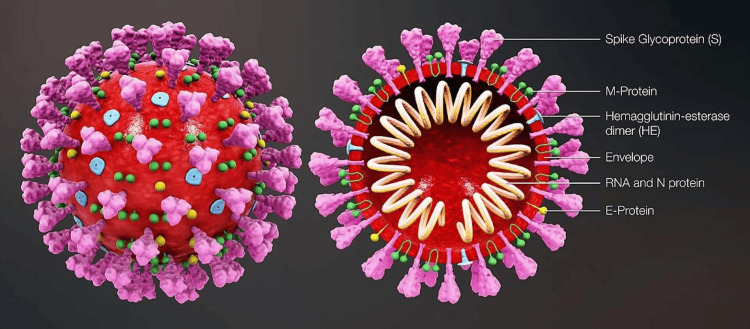
Structure of severe acute respiratory syndrome coronavirus 2 (SARS-CoV-2) M-protein: membrane protein; RNA: ribonucleic acid; N-protein: nucleocapsid protein; E-protein: envelope protein Image Source: Scientific Animations (CC BY-SA 4.0)

SARS-CoV-2 has a diameter ranging from 60-140 nm, with distinctive spikes giving the appearance of a solar corona (Figure 3). The genome size of SARS-CoV-2 is about 30 kb. Bats are likely a natural reservoir for SARS-CoV-2, with a hypothesis that humans are infected via an intermediate host such as the pangolin [73].

The four main structural proteins responsible for the virion assembly and suppression of the host immune response include the envelope protein, membrane protein, nucleocapsid, and spike protein [74]. The spike protein plays a significant role in the host entry of SARS-CoV-2. It consists of two subunits: the S1 subunit, binding to the host ACE-2 receptor, and the S2 subunit, mediating the membrane fusion [75].

ACE-2 receptors are expressed abundantly on type II alveolar epithelial cells and also by the myocardial cells, enterocytes from the ileum, upper esophagus, urothelial cells of the bladder, and proximal tubular cells of the kidney. Upon binding to host ACE-2 through the receptor binding domain (RBD), the S1 subunit undergoes cleavage propelled by the host membrane protein TMPRSS2. This activates the S2 subunit, enabling the fusion of viral and host lipid bilayers, releasing the viral ribonucleoprotein into the host cell, leading to viral replication [76].

**Figure 4:**
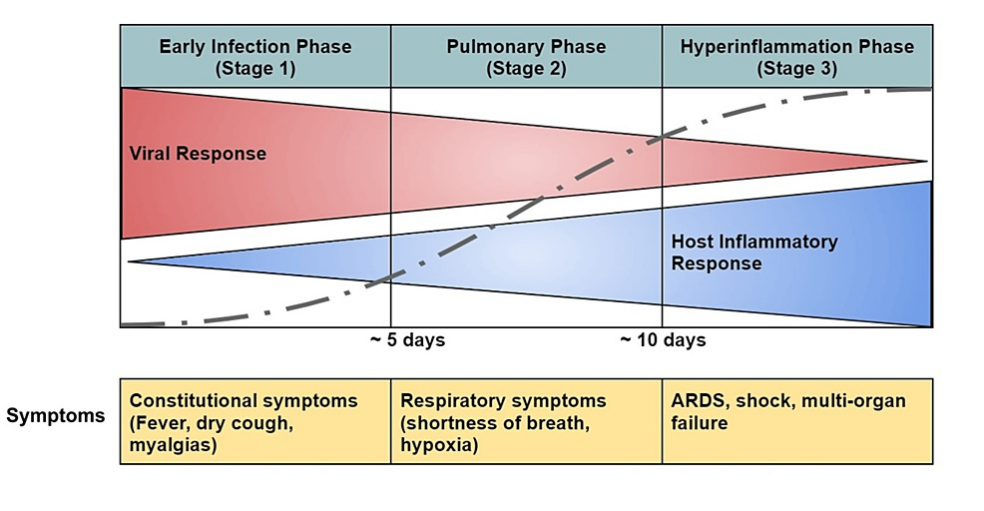
Different phases of coronavirus disease 2019 (COVID-19) progression ARDS: Acute respiratory distress syndrome Image Source: Marik et al., 2021 [77]; Open Access CC BY-NC 4.0 Deed

SARS-CoV-2 predominantly affects the respiratory and vascular systems, thus making COVID-19 a disease mainly of the pulmonary and vascular systems. Three phases based on the severity of the disease have been postulated: early infection phase (stage 1) comprising of viral replication with mild symptoms, pulmonary phase (stage 2) leading to predominantly respiratory symptoms from the host immune response, and finally, the hyperinflammation phase (stage 3) leading to widespread inflammation and tissue injury (Figure 4) [77].

Early Infection Phase (Stage 1)

During this stage, the viral RNA replicates upon entry into the pulmonary parenchyma, leading to mild constitutional symptoms consisting of fever, headaches, myalgias, dry cough, and sore throat. Patients limited to this stage of COVID-19 have excellent prognosis and recovery.

Pulmonary Phase (Stage 2)

In this stage, the continued viral replication and pulmonary inflammation can lead to the development of viral pneumonia and, in severe cases, hypoxia, needing hospitalization.

Hyperinflammation Phase (Stage 3)

About 30% of the patients from the early infection and pulmonary phase progress to this stage [78]. The hallmark of this stage is significant elevations in inflammatory cytokines and biomarkers interleukin (IL)-2, IL-6, IL-7, macrophage inflammatory protein 1-α, granulocyte colony-stimulating factor, tumor necrosis factor (TNF)-α, ferritin, C-reactive protein (CRP), and D-dimer. In severe disease, high levels of cytokines, IL-6, and TNF-α are released, leading to a phenomenon called “cytokine storm,” leading to widespread inflammation [79]. Multiple mechanisms have been proposed for the increased vascular permeability with the resultant pulmonary edema. First, there is direct viral invasion and disruption of the endothelial cell membranes leading to endotheliitis. Second, there is widespread vascular thrombosis with microthrombi and the potential to lead to disseminated intravascular coagulation (DIC). Thirdly, the dysregulated renin-angiotensin-aldosterone system (RAAS) allows increased viral attachment to ACE-2 receptors and increased vascular permeability due to the activation of the kinin-kallikrein system, leading to the release of bradykinin, a potent vasodilator. Lastly, the surge in the inflammatory response leads to enhanced contraction of epithelial cells, leading to cellular edema and disruptions of the tight intercellular junctions. The role of the virus binding to a toll-like receptor (TLR) has been proposed in mediating lung inflammation and fibrosis [78,80-82].

Systemic, widespread inflammation is evident with the release of troponin, and N-terminal pro-B-type natriuretic peptide can also be elevated, in some cases, leading to viral myocarditis [83]. Secondary hemophagocytic lymphohistiocytosis has been reported in advanced diseases [84]. Progressive disease can lead to acute respiratory distress syndrome (ARDS), shock, and cardiopulmonary collapse.

Histopathology

Autopsy studies of patients deceased from severe COVID-19 have provided valuable insights into the myriad of histopathological findings described below according to the organ systems.

Respiratory System

**Figure 5:**
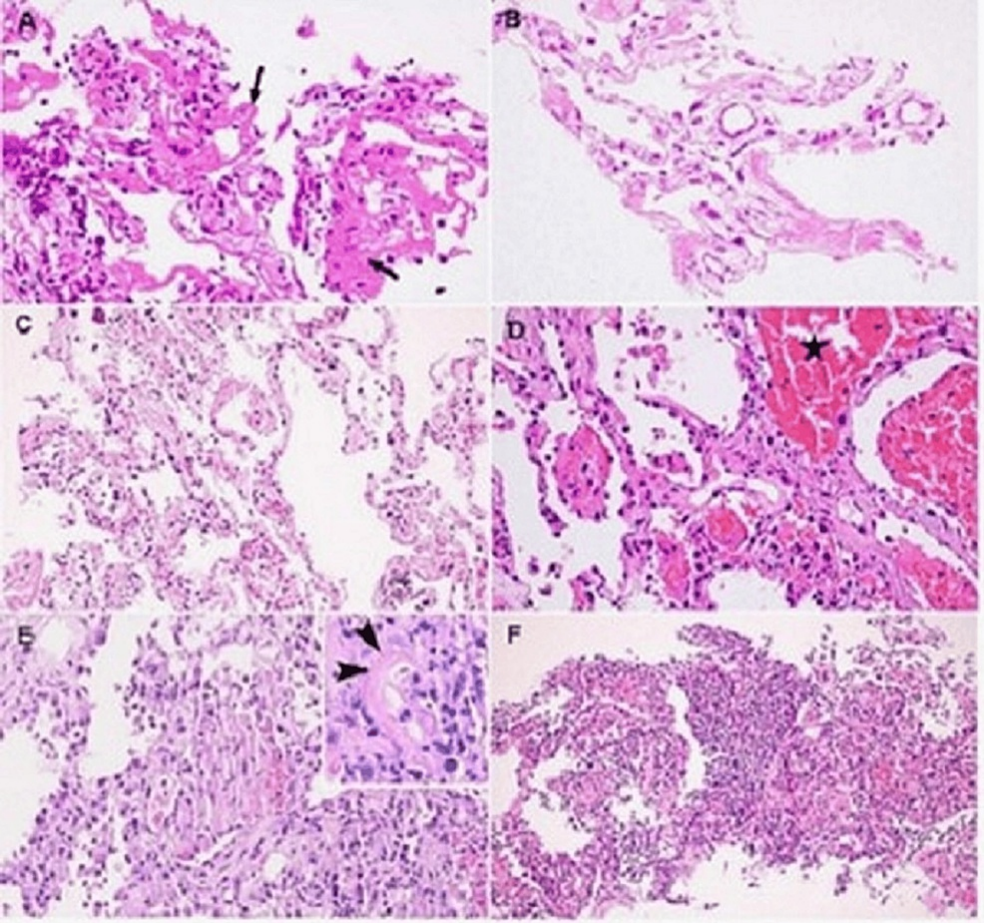
Histological changes from COVID-19 in lungs. (A) Alveolar injury with desquamation and hyaline membrane formation (arrow); (B) No evidence of inflammatory cellular infiltration; (C) Interstitial thickening; (D) Intra-alveolar hemorrhages (asterisk) and the presence of fibrin plugs; (E) Interstitial thickening due to AT2 cell hyperplasia and alveolar infiltration by inflammatory cells with fibrinoid necrosis noted; (F) Intra-alveolar infiltration with neutrophils suggestive of bacterial superinfection COVID-19: coronavirus disease 2019; AT2: alveolar type 2 Image Source: Tian et al., 2020 [86]; Open Access

Macroscopic findings include pulmonary congestion with areas of hemorrhagic necrosis [85]. In an autopsy study of 68 consecutive patients with COVID-19, diffuse alveolar damage was present in 87% of patients. Airway inflammation, alveolar type 2 pneumocyte (AT2 cell) hyperplasia, and the presence of hyaline membranes in the alveolar areas were noted in both short-term and long-term disease; however, AT2 cell hyperplasia and interstitial fibroblastic proliferation were typically seen in patients with longer duration of illness (Figure 5). Extensive pulmonary thromboembolism was noted in 42% of the lungs [86]. In another study Borczuk et al., an autopsy of 10 COVID-19 patients showed the presence of pulmonary thromboembolism in 89% of the lungs studied, abundant with both platelet-predominant and fibrin-predominant thrombi in the vasculature of the alveolar septa [87].

Gastrointestinal System

Liver: Hepatic steatosis appears to be the predominant finding, with focal necrosis in the periportal region [88]. Biliary plugs in the small-sized bile ducts have been observed. Nodular proliferation and periportal inflammation with lymphocytic infiltration are also seen [85].

Gastrointestinal tract: Endoscopic studies have shown no significant damage to the stomach, duodenum, and rectum mucosa. Infiltrating plasma cells lymphocytes were noted abundantly in the lamina propria of the stomach, duodenum, and rectum. Interestingly, viral proteins stain positively in the cytoplasm of the gastric, duodenal, and rectal glandular epithelial cells, excluding the esophageal epithelium [89].

Urinary and Reproductive Systems

Kidneys: Microscopic changes range from diffuse proximal tubular injury with the loss of brush-border epithelium vacuolar degeneration (non-isometric in most cases) to necrosis, as described in an autopsy study of 26 patients with COVID-19. Electron microscopic (EM) examination showed tubular epithelium and podocytes with viral particles bearing distinctive spikes [90].

Testes: In a post-mortem study of testes in 11 male patients deceased from COVID-19 infection, all were noted to show interstitial edema and congestion, tubular basal membrane thickening, strong expression of vascular cell adhesion molecules in the blood vessels, and diminished number of Leydig and Sertoli cells with associated decreased spermatogenesis. EM showed four cases with viral particles in the cytoplasm of Sertoli and Leydig cells, spermatids, fibroblasts, endothelium, and epithelial cells of the rete testis [91].

Nervous System

Brain: An autopsy study to determine the neuropathological findings in 18 patients revealed hypoxic-ischemic injury in all cerebral and cerebellar regions by microscopic exam. No thrombi or evidence of vasculitis was noted. No abnormalities were noted in the olfactory bulbs or tracts. Immunohistochemical analysis was negative for cytoplasmic viral staining [92].

Cardiovascular System

Endomyocardial biopsies of five patients with COVID-19 infection showed myocarditis with necrosis of the myocytes along with granulation tissue and fibrosis along the periphery of necrosis, as noticed after an infarction. Immunohistochemical analysis showed significant intramyocardial inflammation [93].

Skin

Histological findings of COVID-19 viral exanthem varied depending on the stage of the illness. Early stages reveal diffuse telangiectasias of small blood vessels, nests of Langerhans cells within the epidermis, perivascular dermatitis, and dense lymphocytic infiltration. Later stages of infection showed intravascular microthrombi in the small dermal vessels [94].

Organ system manifestations

**Figure 6:**
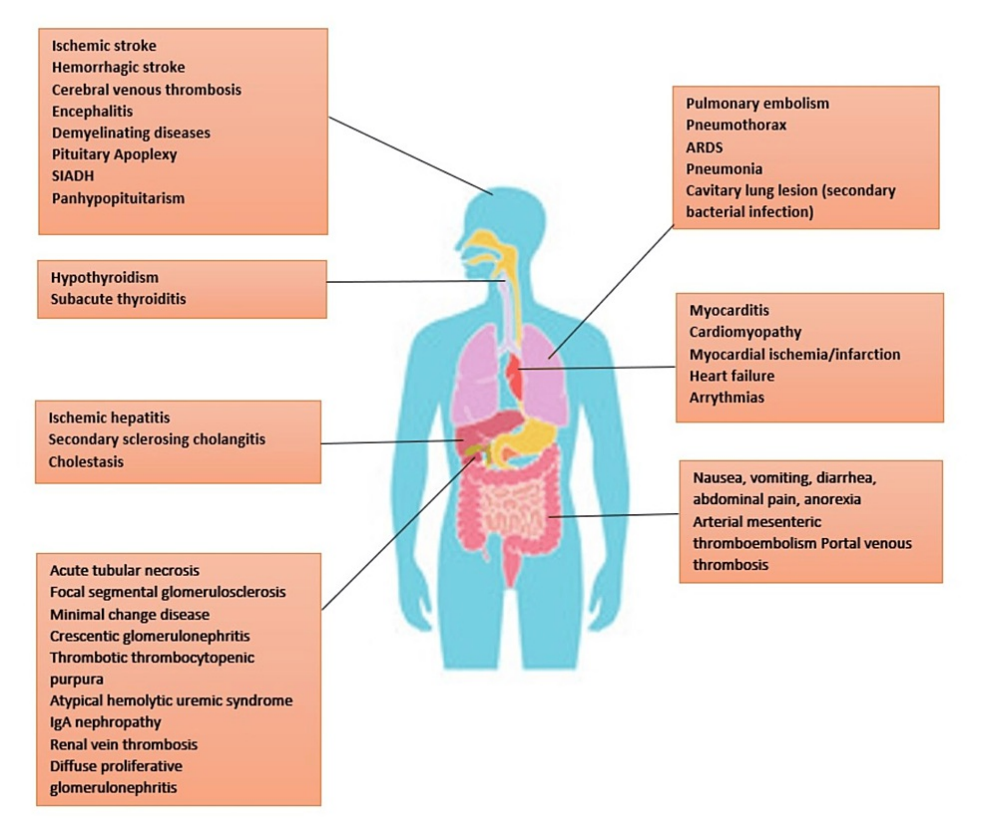
Organ system manifestations of COVID-19 SIADH: syndrome of inappropriate anti-diuretic hormone secretion; IgA: immunoglobulin A; ARDS: acute respiratory distress syndrome; COVID-19: coronavirus disease 2019

SARS-CoV-2 has been associated with widespread disease involving all organ systems. The most devastating effects are seen in the lungs; however, other vital organs are also affected, causing a variety of manifestations. Figure 6 depicts the various organ manifestations.

Cutaneous Manifestations

Various manifestations have been reported from different parts of the world. Most commonly reported presentations include petechia, purpura, ecchymosis, maculopapular rash, COVID-19 digits with ischemia, vesicular eruptions, and chilblain-like rash/lesions [95-98]. Some of the common presentations include COVID toes, which present as erythematous to purplish lesions over toes and fingers, usually self-resolving [96], with some cases progressing to digital ischemia, which is thought to be due to thrombosis and complement deposition, a type 3 delayed hypersensitivity reaction [98]. Livedoid pattern rash is skin mottling with reticular discoloration on the trunk and extremities [96]. Urticarial presentation is likely IgE-mediated type 1 hypersensitivity immune response to viral antigens/drug haptens [95,96,98]. 

Cardiac Manifestations

Various cardiac presentations are seen in COVID-19 patients, including myocarditis, cardiomyopathy, heart failure, myocardial ischemia/infarction (7.2%), arrhythmias (18.7%), and shock (8.7%), and arterial or venous thromboembolism [99-101].

Mechanisms of injury are considered to be due to the direct viral invasion of cardiomyocytes or vascular endothelium, systemic inflammation, cytokine storm causing myocardial injury, supply-demand mismatch due to hypoxemia, and vascular injury causing coagulopathy [98,100]. Interstitial fibrosis in the myocardium is considered to be caused by the suppression of mothers against decapentaplegic (SMAD) pathway activation by tumor growth factor beta (TGF-b) signaling in COVID-19 infection. Patients with COVID-19 are reported to have a lower survival rate when associated with higher NT-proBNP levels (>88.64 pg/ml) [98].

Hematological Manifestations

Different hematological manifestations reported worldwide include thrombocytopenia, increased D-dimer, prolonged activated partial thromboplastin (aPTT), prolonged prothrombin time (PT), low fibrinogen levels, increased serum ferritin, increased lactate dehydrogenase, arterial and venous thrombosis, elevated IL-6, and elevated CRP [102,103]. Sporadic case reports of inflammation related to COVID-19 infection, leading to hemophagocytic lymphohistiocytosis(HLH), have been reported [104]. Increased COVID-19-related complications and in-hospital mortality are reportedly noted in patients with higher D-dimer levels [102]. Disseminated intravascular coagulation (DIC) has also been reported [105].

Mechanisms of injury are considered to be viral entry through adhesion to ACE-2 receptors on endothelial cells, viral replication leading to inflammatory cell infiltration, endothelial apoptosis, and microvascular prothrombotic events. Development of acute procoagulant response to COVID-19 infection due to the high concentration of pro-inflammatory cytokines and chemokines leads to elevated levels of factor VIII, von Willebrand, and fibrinogen, which in turn increases the risk of thrombosis [102].

Renal Manifestations

Diverse renal manifestations reported worldwide include acute tubular necrosis (ATN), collapsing focal segmental glomerulosclerosis, albuminuria, hematuria, electrolyte derangements [98], minimal change disease, crescentic glomerulonephritis, thrombotic thrombocytopenic purpura (TTP), atypical hemolytic uremic syndrome (aHUS), IgA nephropathy, renal vein thrombosis, diffuse proliferative glomerulonephritis, acute kidney injury [106-110].

Mechanism of injury noted from autopsy studies revealed evidence of viral infection in innate monocytes, macrophages, NK (natural killer) cells, evidence of viral replication in renal parenchymal cells, which was thought to cause tissue damage in the form of ATN, and glomerulosclerosis [111]. Other suggested mechanisms include hypoxia, hypoperfusion of kidneys causing ATN, immune complex deposition of viral particles in the kidneys [109], and induction of a proinflammatory state [98].

Pulmonary Manifestations

Pulmonary embolism (PE), pneumothorax, ARDS, and pneumonia are the well-known reported presentations during acute infection [103,112]. Sporadic case of the secondary bacterial process along with COVID-19 infection causing cavitary lung lesion [113] has been reported. Pneumothorax due to barotrauma from ventilation, as well as spontaneous pneumothorax due to damage to the alveolar wall, has been reported with a median duration of nine days since the start of symptoms, with a mortality rate of 36% [103,114]. Spontaneous pneumomediastinum in a non-ventilated patient has also been reported [115]. An autopsy study showed that massive PE can occur in initially asymptomatic COVID-19 patients [103,116]. In an intensive care unit, COVID-19 patients, compared to influenza patients, had a three times higher incidence of PE [103,117]. The mortality rate in COVID-19 patients is increased with coexistent PE [103,118].

Gastrointestinal Manifestations

About 5-50% of patients with COVID-19 infection develop gastrointestinal symptoms such as diarrhea, nausea, vomiting, anorexia, and abdominal pain [119]. In 5% of patients, gastrointestinal symptoms precede respiratory symptoms, and a high index of suspicion is required for a timely diagnosis in these patients [120]. The ACE-2 receptors are highly expressed in the stratified epithelium of the esophagus, stomach, small intestine, and colon. The mechanisms of gastrointestinal injury in patients with COVID-19 infection include direct infection of the gastrointestinal cells by SARS-CoV-2, cytokine storm, and gastrointestinal damage caused by lung infection by an effect known as “gut-lung axis” in which the damage to the respiratory tract affects the digestive tract via immune regulation [89,121-125].

In rare instances, patients with COVID-19 infection develop ischemic gastrointestinal complications, which can be fatal. In a systematic review of 31 patients, 29% presented with arterial mesenteric thromboembolism, 19.3% with portal venous thrombosis, 64.5% required laparotomy with bowel resection, and the overall mortality was 38.7% [126]. Examination of the small bowel with mesenteric thrombosis revealed prominent endothelium, direct viral invasion of endothelial cells, and diffuse endothelial swelling with mononuclear cell infiltration [124]. Patients with severe COVID-19 infection and receiving inotropic agents can develop nonocclusive colonic ischemia due to severe vasoconstriction and reduced mesenteric blood flow [127].

Hepatic Manifestations

A systematic analysis of 11 studies with 2,542 patients showed increased aspartate aminotransferase (AST) and/or alanine aminotransferase (ALT) (25%) and bilirubin (3%) in patients with COVID-19 infection [128]. The mechanisms of liver injury in patients with COVID-19 infection are direct cytopathic damage by SARS-COV-2 binding to cholangiocytes expressing ACE-2, cytokine storm, systemic inflammatory response syndrome (SIRS)-induced cholestasis, ischemia hypoxia reperfusion injury, and drug-induced liver injury [119,125,129,130].

Ischemic hepatitis rapidly increases serum aminotransferases and may be secondary to septic shock, COVID-19-related myocarditis, and ventilator complications [131]. Liver biopsies of patients who died of complications of COVID-19 demonstrated congestion and centrilobular ischemic necrosis in 78% and 40% of cases, respectively [132]. Patients with severe COVID-19 infection can develop secondary sclerosing cholangitis of critically ill patients (SSC-CIP) because of reduced perfusion, hypoxia, and recurrent inflammatory stimuli on the biliary epithelium [133].

Neuropsychological Manifestations

The various neurological manifestations of COVID-19 infection include cerebrovascular disease, encephalopathy, damage from the immune response, neurodegenerative and demyelinating disorders, seizures, headaches, dizziness, and mental disorders.

Cerebrovascular disease includes ischemic stroke, intracranial hemorrhage (ICH), and cerebral venous thrombosis. The incidence of ischemic stroke in patients with COVID-19 infection is 5-6.92% and predominantly seen in men with a median age of 63 [134-136]. Ischemic strokes have been reported in younger patients (age <50) without cardiovascular risk factors before the onset of COVID-19 symptoms and with systemic thrombosis in a quarter of these patients [137-139]. ICH is reported in 2.66% of patients with COVID-19. ICH is suspected to be due to vascular endothelial damage and cerebral microhemorrhages more commonly seen in the brainstem [140,141]. Cerebral venous thrombosis (CVT) is a rare neurological manifestation secondary to the hypercoagulable state caused by COVID-19 infection, and low molecular weight heparin is recommended as the first-line treatment [142,143]. In hypertensive encephalopathy, the viral receptors occupy ACE-2 receptors, and the chronic depletion of these receptors increases the risk of angiotensin II (Ang II) dependent hypertension [144]. This leads to blood-brain barrier disruption, cerebral hyperperfusion, and hypertensive encephalopathy [145].

Abnormal immune and inflammatory reactions cause nervous system damage. In acute necrotizing encephalopathy (ANE), cytokine storm in severe COVID-19 infection can result in disruption of the blood-brain barrier and brain necrosis evidenced by bilateral hemorrhagic rim-enhancing lesions in the thalamus [146-148]. Guillain-Barré syndrome (GBS) in COVID-19 is more prevalent in the elderly and males. Patients experience symptoms of COVID-19 for 5-14 days before the onset of paresthesia, lower limb, and facial weakness [149]. Myalgia and myositis have been noted following COVID-19 vaccination [150]. There is no evidence of acceleration of multiple sclerosis (MS), Alzheimer's disease, and Parkinson’s disease, in patients with COVID-19 infection [151]. Few patients develop seizures due to hypoxia, severe inflammatory state, metabolic derangements, and COVID-19-induced brain damage, lowering the seizure threshold [152-154]. Headache and dizziness are the most common neurological symptoms of COVID-19 infection occurring in 6-25% and 8-9% of patients, respectively [155].

The incidence of anxiety, depression, and post-traumatic stress disorder (PTSD) after COVID-19 infection is 17.39-34.7%, 28.5%, and 96.2%, respectively [134,156]. Due to social isolation and other risk factors, there is an increase in mood disorders, drug abuse, and suicidal tendencies [157,158].

SARS-CoV-2 spreads to the nervous system via the olfactory route through the cribriform plate, the trans-synaptic route via retrograde axonal transport from the peripheral nerves, the leukocytic route via migration of viral particles across the blood-brain barrier, and hematogenous route. The mechanisms causing neurological damage in COVID-19 infection are direct viral binding to the neurons and glial cells, damage to the blood-brain barrier and endothelial system, cytokine storm, effects of hypoxia, post-infectious autoimmune effects via the cellular immunity and autoantibodies, and coagulation disorders [140,159]. In a systematic review of 22 studies, the frequency of COVID-19-associated anosmia, hyposmia, ageusia, and dysgeusia was 55%, 40%, 41%, and 31%, respectively [160]. The smell and taste disorders last for about 10 days in patients with a mild infection, and complete resolution is reported in 89% of patients after four weeks [161].

The olfactory neuroepithelium comprises olfactory sensory neurons, sustentacular cells, microvillar cells, Bowman glands, and basal cells [162]. The olfactory receptors and impulses detect the airborne chemicals transmitted to the olfactory bulb, where the second-order neurons send projections to the primary olfactory cortex in the temporal lobe [163]. It is suspected that SARS-CoV-2 infection causes anosmia by damaging the sustentacular cells, which are essential for the maintenance and normal function of olfactory cilia, and long-lasting anosmia in a small number of patients is due to extensive destruction of the sensory epithelium [164]. The epithelial taste receptors arise from the basal layer of the taste bud and are embedded in the tongue epithelium, palate, and pharynx. The taste cells are connected to the gustatory centers via the cranial nerves VII, IX, and X. Neuropilin-1 (NRP-1) expressed in gustatory nerve fibers and the inflammatory cytokine storm are suspected to play a role in the loss of taste sensation [163].

Ocular Manifestations

Conjunctivitis is the most common manifestation, with a prevalence of about 7%, and can manifest early or late (after 10-13 days) [165]. There are rare case reports of keratitis, pseudomembranous keratoconjunctivitis, conjunctival follicular reaction, episcleritis, hemorrhagic and pseudo-hemorrhagic conjunctivitis [166,167]. Eyelid manifestations include blepharitis, eyelid edema, eyelid dermatitis, and meibomian orifice abnormalities [168]. Retinal manifestations are secondary to the expression of ACE-2 receptors in the retina and thromboembolic complications. The retinal vascular manifestations include: (i) Central retinal vein occlusion, associated with impaired vision, while some are asymptomatic. Fundus examination of these patients demonstrated retinal hemorrhages, pan-retinal fern-like whitening, macular edema, and dilated and tortuous vessels, (ii) Central retinal artery occlusion, (iii) Acute macular neuro retinopathy, (iv) Paracentral acute middle radiculopathy, (v) Vitritis, and (vi) Acute retinal necrosis [168-171]. Chorioretinitis is suspected to be due to the inflammatory effect of COVID-19 infection. Neuro-ophthalmic manifestations include optic neuritis, Miller-Fisher syndrome, and cranial nerve palsy [168]. Rhino-orbital cerebral mucormycosis is the most common orbital manifestation resulting from an impaired immune system, use of corticosteroids, and underlying conditions such as diabetes and renal failure [172,173]. Orbital cellulitis is also reported in some patients.

COVID-19-associated endocrinopathies

The COVID-19 pandemic, which has caused catastrophic mortality and morbidity, is still significantly negatively impacting global healthcare systems. It becomes more and more evident that this novel respiratory virus's effects go beyond the respiratory system as time passes and our knowledge of it deepens. This has led to much curiosity about how COVID-19 affects the endocrine system, along with several case reports of thyroid and pituitary disruption in these patients. To inform us of proper research and effective management, this review provides an overview of the data examining the implications of COVID-19 on each endocrine axis.

Thyroid

In general, thyroid problems are not linked to the severity of COVID-19, nor are they linked to mortality or susceptibility [174]. On the other hand, abnormal thyroid function tests were often detected in hospitalized COVID-19 patients. These abnormal thyroid function tests were characterized by low thyroid-stimulating hormone (TSH) levels and free thyroxine (fT4). Seven percent of COVID-19 patients who were hospitalized were found to have non-thyroidal sickness syndrome (NTIS), which is defined by low levels of free triiodothyronine (fT3), high levels of reverse T3, low or normal levels of TSH, and low levels of free thyroxine (fT4) [175]. Low levels of fT3 at the time of admission were linked to worsening clinical conditions [176], increased disease severity, and death [177,178]. According to Chen et al., the risk ratio for mortality in COVID-19 individuals with NTIS is 11.64 (95%CI: 4.88-27.78) [177]. This was compared to patients who did not have NTIS. Even though both symptomatic and asymptomatic cases of subacute thyroiditis (SAT) due to SARS-CoV-2 infection were recorded, symptomatic SAT due to COVID-19 was an extremely unusual outcome. The SAT linked to COVID-19 was very comparable to the traditional SAT, and there was no increase in the frequency of SAT recorded during the pandemic. The information that is now available shows that thyroid function tests return to normal once recovery has taken place and that SARS-CoV-2 does not have any long-term influence on thyroid functioning [179].

In general, COVID-19 patients, especially those in the ICU, have a high prevalence of thyroid dysfunction, likely an indicator of NTIS. Although NTIS is linked to mortality, thyroid tests typically return to normal after symptoms subside. COVID-19 individuals with preexisting thyroid issues should be treated following the guidelines for addressing such conditions [176-179].

Pituitary

Pituitary disorders are uncommon, making obtaining data on the COVID-19 course in such patients difficult. However, hypopituitarism does not appear to affect COVID-19 results. COVID-19 may be linked to new-onset hypopituitarism via pituitary apoplexy (PA) and hypophysitis.

PA is a clinical and surgical emergency syndrome that is caused by a rapid hemorrhage and blood infarction of the pituitary gland, usually inside a pituitary macroadenoma. It is an acute condition that requires immediate medical attention. Patients often report experiencing abrupt onset and severe headaches, visual abnormalities, and ocular palsy as their primary symptoms [180]. These symptoms are caused by the tumor's hemorrhagic and necrotic mass crushing the surrounding optic structures and extending into the cavernous area.

Since SARS-CoV-2 can produce thrombocytopenia, coagulopathy, and platelet dysfunction and has neural tissue tropism owing to ACE-2 expression in cerebral vascular endothelium, COVID-19 could be a possible precipitating risk factor for pituitary apoplexy. This is because COVID-19 has been shown to cause apoptosis of the pituitary gland [181].

As electrolyte imbalances, in general, are seen most frequently in hospitalized patients, hyponatremia has been reported in 20-60% of patients who have had COVID-19. The pathophysiology of hyponatremia in COVID-19 is not well known; however, the syndrome of inappropriate antidiuretic hormone secretion (SIADH) and hypovolemia are considered to be contributing factors. Although hypernatremia is less uncommon than hyponatremia (5%), it is noticed in COVID-19 patients, particularly in the ICU, and is associated with an unfavorable outcome [182].

Based on the above evidence, COVID-19-related prothrombotic and endothelial systemic disease may trigger pituitary apoplexy risk factors, particularly in individuals afflicted by pituitary adenomas. Therefore, in both specialist pituitary tumor centers and general medical settings, there has to be a greater awareness of this consequence, particularly in patients prone to developing it (macroadenoma on dopaminergic medications, anticoagulant treatment).

Patients with COVID-19 who report new-onset headaches and/or neuro-ophthalmological symptoms should be tested at the pituitary gland level (pituitary apoplexy, hypophysitis). Because hyponatremia is so frequent in hospitalized COVID-19 patients, it is essential to check their blood salt levels regularly [183].

Adrenal

COVID-19 has been linked to newly developed primary adrenal insufficiency. Bilateral adrenal hemorrhagic or non-hemorrhagic infarction have been seen in such patients despite no obvious infarction or thrombi in the adrenal glands documented in postmortem studies [88]. Nearly a quarter of patients with moderate to severe COVID-19 had evidence of acute adrenal infarction on their first chest CT scans, 88% of these cases being bilateral. Patients with adrenal infarction were more likely to be admitted to the ICU, stay in the hospital longer, and survive, but there was no data on biochemical/clinical hypocortisolism [184].

COVID-19 did not exhibit critical illness-related corticosteroid insufficiency (CIRCI), a compromised hypothalamic-pituitary axis (stress) response during critical illness. Instead, the scant evidence suggests that severe cases of COVID-19 are associated with significantly higher cortisol levels in patients compared to those with milder illnesses [185]. Similarly, within 48 hours of admission, COVID-19 patients had greater blood cortisol concentrations compared to non-COVID-19 controls, and this was associated with a decreased likelihood of survival [186]. However, glucocorticoids, especially dexamethasone, help severely or critically sick COVID-19 patients, probably because of their anti-inflammatory effects.

Histopathological investigations have been the primary source of information regarding the potential effects of COVID-19 on the adrenal glands. The human adrenal cortex contains TMPRSS2 and ACE2. Thus, animal and human studies have demonstrated SARS-CoV-2 expression and replication in the adrenal glands [187].

Chronic insufficient cortisol production from several causes characterizes adrenal insufficiency. Patients with primary adrenal insufficiency, primarily Addison disease, and secondary adrenal insufficiency should be regarded as at risk for being impacted by COVID-19, as indicated in a recent statement made by the European Society of Endocrinology. Adrenal crisis exacerbations have been linked to an increased risk of infection for two reasons: first, because of the increased risk of infections reported in adrenal insufficiency patients overall [188], likely due to their inefficient innate immune response, and second, because of the increased mortality rate observed facing respiratory infections [189].

Overall, our knowledge is limited on how COVID-19 affects the adrenal gland, how the adrenal gland responds to COVID-19, and how COVID-19 progresses in people with adrenal diseases. However, the patients examined at admission and found to have hypercortisolemia rather than CIRCI were not re-evaluated later during their hospital stay, which might have resulted in different outcomes. Currently, many studies are looking at the effects of long COVID, including hypocortisolism post-COVID-19.

Reproductive System

Since the beginning of the pandemic, researchers have known that male sex is associated with increased COVID-19 severity and mortality but not susceptibility, highlighting the role of sex in COVID-19 outcomes. Overall, males have a 1.4-fold higher chance of dying of COVID-19 than females [190], but postmenopausal women and men have similar survival rates.

Menstrual irregularities in women and orchitis or epididymitis in men in up to 20% of patients [191] suggest that COVID-19 may affect the reproductive system. Additionally, 6.9% and 1.4% of semen samples were positive for SARS-CoV-2 during the infection and recovery periods, respectively [192,193].

Research revealing low testosterone, oligozoospermia, and immobile sperm in SARS-CoV-2-infected males prompted concerns regarding the effect of COVID-19 on male fertility [194]. Immediate and delayed consequences include testicular discomfort, epididymal-orchitis, or isolated orchitis, which have all been reported by individuals with COVID-19 [195], consistent with histological results. Similarly, 10.9% of patients in a study with acute COVID-19 reported testicular discomfort [196]. At one week to one month post hospitalization, ultrasonography showed orchitis or epididymal-orchitis in more than 20% of 142 males with acute COVID-19 infection, with the risk of epididymal-orchitis rising with COVID-19 severity and advancing age [197]. At a median of 80 days after infection, sperm concentration and total sperm count were lower in 55 male patients who had recovered from COVID-19 compared with healthy controls of the same age [198].

There was a correlation between pregnancy and an increased chance of severe COVID-19, which ranged from a 2.5-fold to a 5-fold increase, and an increased risk of mortality, which was 1.7-fold higher. There is insufficient evidence to conclude whether pregnancy is also linked to COVID-19 susceptibility [199]. The usual risk variables linked with the severity of COVID-19 were significant in pregnant women. These risk factors included a higher body mass index (BMI) and pregestational comorbidities such as diabetes and older maternal age. Furthermore, COVID-19 has the potential to influence maternal and perinatal outcomes. The relative risks of pre-eclampsia/eclampsia, premature delivery, and fetal distress were found to be 1.76 (95%CI 1.27-2.43), 1.59 (95%CI 1.30-1.94), and 1.70 (95%CI 1.06-2.75), respectively [200]. On the other hand, research showed an elevated risk for stillbirth but not for premature birth [201]. Immunoglobulin M positive in cord blood was found, even though it is thought that vertical transmission of SARS-CoV-2 infection from mothers to neonates is highly rare [202].

Pancreas

During the SARS epidemic, hyperglycemia in individuals not previously known to be diabetic was recorded. During their inpatient hospitalization, 51.3% of nondiabetic patients diagnosed with SARS met diagnostic criteria for diabetes [203]. Similarly, reports appeared of patients presenting with ketosis, new-onset hyperglycemia, and new diagnoses of diabetes [204]. Patients with type 1 or type 2 diabetes were shown to have an elevated risk of death following COVID-19. It has been demonstrated that SARS-CoV-2 can infect and proliferate in human endocrine pancreas cells [205], and SARS-CoV-2 viral RNA has been found in the cells of COVID-19 patients at autopsy. In the microvasculature of the pancreas, both the ACE-2 receptor and the TMPRSS2 protein have been identified as being present [206].

There have been reports of people developing type 1 diabetes for the first time after COVID-19 [207], with some maintaining a negative islet cell autoantibody status [208]. Therefore, the presence of autoantibody-negative diabetes that requires insulin after COVID-19, in conjunction with the histological results, implies that, at least in some people, COVID-19 may be linked with islet-cell functional impairment or destruction. This is the case even if autoantibodies were not detected in the patients.

Ketoacidosis can develop when inadequate insulin is released from the pancreas to fulfill the body's glycemic demands. This condition is most commonly seen in people with type 1 diabetes, which is caused by the death of beta cells by the immune system. On the other hand, ketoacidosis has been recorded in individuals with type 2 diabetes mellitus (T2DM) who were treated with COVID-19. However, it is also conceivable that this is a consequence of the high insulin resistance reported in patients with COVID-19, leading to beta cell failure [209]. In most instances, ketonemia appeared to be related to insulinopenia [210]; however, it is also possible that this is a consequence of the significant insulin resistance observed in patients with COVID-19.

Obesity

It should not come as a surprise that obesity has emerged as a major problem during the COVID-19 pandemic, given the global prevalence of obesity 13% of the adult population and the well-documented detrimental impacts that obesity has during serious diseases [211]. Obesity is linked to increased COVID-19 severity and potentially even death, according to a substantial amount of clinical research that has been conducted, a finding that is corroborated by Mendelian randomization studies [212]. It has been reported that patients infected with SARS-CoV-2 had a higher overall prevalence of obesity than the general population. This suggests that obesity may make people more susceptible to infection with SARS-CoV-2. It has also been suggested that higher visceral adipose tissue can be used as a predictor of the severity of COVID-19, in addition to BMI [213].

In addition to the influence obesity has on the prognosis of COVID-19, "stay home" tactics used during the pandemic resulted in physical inactivity, stress, sleep deprivation, and bad eating behavior, all of which might lead to an increase in obesity in persons regardless of whether or not they were infected with SARS-CoV-2. Older people may be at a greater risk of developing sarcopenic obesity than younger people because of the hormonal and immunological changes associated with aging [214].

There is a correlation between COVID-19 severity and likely mortality in people of all ages who are obese, both men and women. There is a need for more research to identify, in particular, whether or not obesity affects the risk of infection and severe COVID-19 and how this occurs.

Recent trends of severity compared to the beginning

WHO nomenclature named coronavirus variants using the letters of Greek alphabets, starting with the Alpha variant, which emerged in 2020. Since the pandemic's start, several notable variants include Alpha, Beta, Delta, and Omicron.

Alpha: The CDC identified Alpha (B.1.1.7) as a variation of concern after its initial appearance in Great Britain in November 2020. According to estimates, the B.1.1.7 lineage was more contagious than the original SARS-CoV-2 strain.

Beta: By the end of 2020, Beta (B.1.351) was first identified in South Africa and quickly spread to other nations. The US did not see Beta frequently. The Beta variant had much higher odds of progressing to severe disease as compared with the Alpha variant [30]

Delta: In late 2020, Delta (B.1.617.2) was discovered for the first time in India; compared to earlier varieties, it was twice as infectious as the prior variants [32]. Hospitalization from Delta was more likely to occur in the unvaccinated. The most well-known of the Delta offshoots was Delta AY.4.2, which is frequently inaccurately referred to as Delta Plus.

Omicron: In the last two years, Omicron and its sub-variants have been the most common SARS-CoV-2 strains in the United States [22]. Several recent Omicron strains in circulation, such as BF.7, XBB, BN.1, and BF.11, are still being studied by experts. Omicron subvariants are thought to be very effective disease carriers, and although researchers are still learning about XBB.1.5, they believe it to be the most transmissible strain to date. One explanation was that the spike protein, which binds to human cells, had more than 30 alterations, many of which are thought to increase the likelihood of infection. The Omicron variant, confirmed to be more transmissible and less virulent than previously circulating variants, has taken over as the dominant strain since 2021 [215,216].

Cases quickly spread to neighboring countries after the initial Omicron strain (BA.1) was found in South Africa and Botswana in late November 2021. By December 2021, Omicron had increased the number of daily cases in the U.S. to over a million. In 2022, many sub-variants, including BA.5, BQ.1, and BQ.1.1, were identified from it. By January 2023, a new Omicron subvariant known as XBB.1.5 was responsible for most infections in the US.

COVID-19 is a complicated illness with symptoms ranging from asymptomatic infection to multiorgan failure. The severity of COVID-19 may alter over time for various causes among the demographics. The introduction of new variants, development, and usage of more effective treatments may impact clinical care and the severity of the disease. The severity of three high-COVID-19 transmission periods has been studied using a variety of surveillance system indicators, including the number of cases, emergency department (ED) visits, admissions, ICU utilization, and deaths.

The total number of cases in the US until May 2023 is 103.37 million. The CDC reviews information from three different surveillance systems and a major healthcare database to evaluate several indicators during three high-COVID-19 transmission periods: December 1, 2020-February 28, 2021 (winter 2020-2021); July 15 to October 31, 2021 (predominance of SARS-CoV-2 B.1.617.2 (Delta)), and December 19, 2021, to January 15, 2022 (Omicron predominance) [67,217].

Higher vaccination coverage, which decreases disease severity [217], lower virulence of the Omicron variant [216,218,219], and infection-acquired immunity are all likely major contributors factors to the less severe COVID-19 disease during the Omicron period than previous periods of high transmission [216,220].

The highest weekly case rate per 100,000 population was 516 (December 2020-February 2021), 354 (July- October 2021), and 1696 (December 2021-January 2022). During the Omicron surge, the total cases and the highest percentage of ER visits was 13.6%, the ratio of total cases to the highest hospital admission was 140K, and the highest weekly death rate per 100,000 population was 5.23% compared to the winter of 2020-2021 period (7.4%, 125K and 7.4 respectively) and Delta (7.2%, 95K, 4.3 respectively) [67].

A maximum of 20.6% of staffed inpatient beds were used for COVID-19 patients during the Omicron period, which is 3.4% and 7.2% higher than the winter of 2020-21 and during the Delta surge, respectively. The use of beds in the ICUs did not rise to the same extent. During the Omicron surge, 30.4% of staffed ICU beds were used for COVID-19 patients, 1.2% higher than during the Delta surge and 0.5% less than during the winter of 2020-21 [67].

Since December 2021, the Omicron variant has caused a notable rise in COVID-19 cases in the US. Although the sudden increase in cases has strained the healthcare system and led to the most COVID-19-related ED visits and hospital admissions since the outbreak, the disease severity seems lower than during prior surges. In addition to decreased rates of ED visits, hospital stays, and fatalities relative to cases seen during the Omicron period, hospitalized COVID-19 patients also had lower levels of severity of illness markers such as ICU admissions, need for mechanical ventilation, length of stay, and in-hospital mortality. The use of vaccine boosters by recommended subgroups and increases in vaccination coverage among eligible individuals [67,217] are two main contributing factors to the apparent decline in disease severity [67]. Another important determinant for less severe disease is infection-acquired immunity [216,220] and the potential for decreased virulence of the Omicron variant [216,218,219].

WHO has now classified XBB.1.5 as a VOI. It is a subvariant of Omicron, reported to be identified in 38 countries [221]. Although this variant is not associated with severe illness, the present vaccines do not induce high neutralization titers against this variant [222].

Evaluation

Detailed history, including duration of symptoms, travel, exposure, pre-existing conditions, and substance abuse, should be taken by the clinician. In the acute phase of the infection, prompt diagnosis using nucleic acid amplification testing (NAAT) and serologic testing must be done.

NAAT

Reverse transcription-polymerase chain reaction (RT-PCR) is considered the gold standard in detecting SARS-CoV-2 infection. With a specificity of nearly 100%, the sensitivity can depend on multiple factors, including sample source, sample adequacy, and time from exposure [74]. NAATs done in the laboratory have a higher sensitivity than point-of-care-based tests. Commercially available NAATs are designed to target multiple virus genomes as opposed to NAATs designed to focus on one target, leading to false negative results when there is a mutation in the genome targeted by the test. SARS-CoV-2 RNA can be detected by NAATs weeks to months after the symptom onset for COVID-19; however, isolation of competent virus beyond 10 days of symptom-onset in mild disease and over 20 days in severe disease is low [223]. Hence, the CDC does not support repeat NAATs within 90 days in asymptomatic patients with previous SARS-CoV-2 infection [224].

Antigen Testing

Antigen-based tests detecting viral antigens have less sensitivity than NAATs but have high specificity. They have a rapid turnaround with a lower cost, making it attractive point-of-care testing, especially in crowded living situations like nursing facilities, dormitories, and correctional facilities. False positive results have been identified when the test is completed without following the instructions, human antibodies like rheumatoid factor interfering with the testing, and used in settings with a low prevalence of COVID-19 infection.

Antibody or Serologic Testing

Antibody-based testing detects SARS-CoV-2 antibodies and is available for use; however, these tests are not recommended for diagnosing SARS-CoV-2 infection since seroconversion can take over 21 days. Serologic testing has been utilized when both NAATs and antigen tests have yielded false-negative results.

Therapeutics

Need for Effective Treatments and Vaccines

The COVID-19 pandemic brought unprecedented challenges to the scientific and healthcare community. It ushered in the need for effective treatments and vaccines that had to be tested in a real-world setting rapidly. The number of clinical trials has increased exponentially since the start of the pandemic, and the number of studies related to COVID-19 is now around 8,700. The currently actively recruiting trials are also numerous, approximating around 2,000 worldwide [225]. Tested therapies are being approved at a rapid rate by regulatory bodies across countries in efforts to stop the spread of the virus and to decrease mortality. Several emergency authorizations have been granted to bring treatments, and global efforts are underway to repurpose drugs to bring treatments to people in an accelerated timeline while awaiting benefits from trials. However, not all trials have been successful. Many of the treatments that may have shown efficacy in earlier trials failed to do so in large randomized controlled trials (RCTs), e.g. baloxavir, marboxil, lopinavir/ritonavir, chloroquine, hydroxychloroquine, interferon β-1a, colchicine, favipiravir, ivermectin, ruxolitinib, tofacitinib, metformin, and convalescent plasma. There is also a growing sense of discomfort and criticism about safety, weak methodology, and the power of these trials, causing disagreements and debates among clinicians. This review will shed light on the large trials that have shown the success of a certain therapy or a lack of efficacy of others up until this cross-section of time. The readers are urged to continuously update their knowledge and follow best practice guidelines in this dynamic and rapidly evolving science.

Importance of Data Sharing

Many institutions, pharmaceutical companies, government agencies, and journals have acknowledged the importance of data sharing in clinical trials. The release of de-identified individual participant data has been particularly emphasized. The European Medicines Agency (EMA) and Health Canada have implemented landmark transparency policies, both of which now post sections of the licensure dossier received by the industry on their websites [226]. Additionally, industry and academic platforms, such as ClinicalStudyDataRequest.com [227], Yale University Open Data Access (YODA) Project [228], and Vivli [229], have been created to facilitate third-party access to trial data and documents. Since 2015, the US Institute of Medicine has endorsed the benefits of sharing clinical trial data, emphasizing that verifying and replicating investigators’ claims were essential to the scientific process [230].

COVID-19 trials overview

COVID-19 Trial Challenges

During the initial phase of the pandemic, enrollment and randomization were difficult due to lockdown and social distancing, but these later improved. Other external factors, such as resource limitations in certain countries and the changing political landscapes, are some of the many hurdles investigators have faced. There have been recommendations to establish committees, incorporate governance to balance power, centralize funding, and engage community stakeholders by certain groups [231,232].

Currently, Alpha (B.1.1.7), Beta (B.1.351), Gamma (P.1), Delta (B.1.617.2), and Omicron (B.1.1.529) are the major variants that are present in the community. The WHO classifies others as VOI, VOHC, and variants under monitoring [233]. The emergence of these variants has changed the standard of care and utilization of new therapies, affecting the outcome of trials and may continue to do so.

Types of Trials

**Figure 7:**
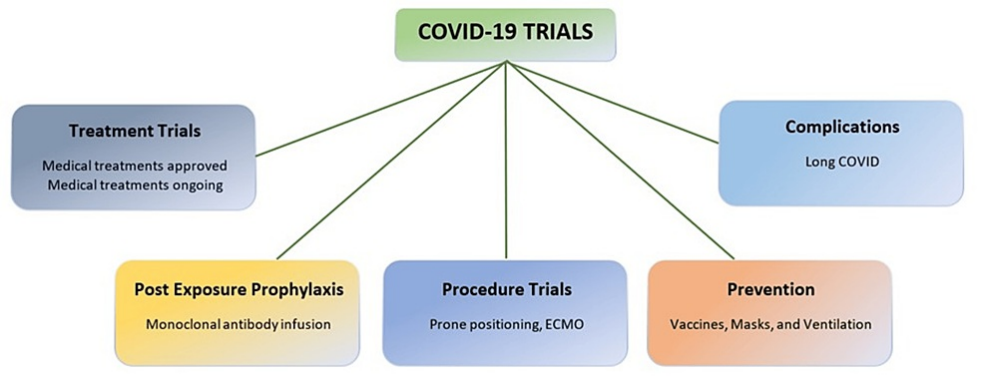
List of various types of COVID-19 trials Covid: coronavirus disease, ECMO: extra-corporeal membrane oxygenation; COVID-19: coronavirus disease 2019

The different types of trials are shown in Figure 7. The trials include: (i) Prevention trials to evaluate vaccines or other interventions to prevent people from getting infected with SARS-CoV-2. These trials typically involve healthy individuals; (ii) Treatment trials to test treatments for people already diagnosed with COVID-19. Treatments may include drugs, monoclonal antibodies, or other therapies; (iii) Diagnostic trials to test new diagnostic tests or procedures for COVID-19. These tests can help diagnose early infection and identify individuals who may be asymptomatic carriers of the virus; (iv) Prognostic trials to identify predictors of disease severity or outcome. They can help identify patients at high risk for severe disease or complications; and (v) Observational studies, which gather data on patients with COVID-19 and help researchers understand how the disease progresses, how different patient populations are affected, long-term complications from the disease, and which treatments are most effective.

COVID-19 made researchers launch trials sooner than earlier. For example, the Randomized Embedded Multifactorial Adaptive Platform for Community-acquired Pneumonia (REMAP-CAP) trial expanded its existing infrastructure to include COVID-19 patients. The Randomised Evaluation of COVID-19 Therapy (RECOVERY) trial used nationwide electronic health record (EHR) data to assess outcomes [234]. Owing to the urgency of the pandemic, several countries implemented fast-track procedures to reduce review processes for trials to be conducted [235]. The rapid enrollment in trials such as RECOVERY, Adaptive COVID-19 Treatment Trial (ACTT), Solidarity, REMAP-CAP, Helping Alleviate the Longer-term Consequences of COVID-19 (HEAL-COVID), Prophylactic Treatment of COVID-19 in Care Homes Trial (PROTECT-CH), PRINCIPLE (Platform Randomised Trial of Treatments in the Community for Epidemic and Pandemic illnesses), COVID-19 Monoclonal Antibody Efficacy Trial-Intent to Care Early (COMET-ICE), Blocking Viral Attachment and Cell Entry with SARS-CoV-2 Neutralizing Antibodies (BLAZE-1) set practice standards for other trials to be conducted in an expedited manner and for phase-2 trials to supply data to phase-3 trials in a prompt fashion. Large pragmatic, innovative trials like RECOVERY allowed multiple treatment arms to be conducted simultaneously. The framework of these ‘Trials Within Cohorts (TWiCs)’ could potentially be used for future research and other endemics. Table 2 shows the large trials conducted in the past few years.

**Table 2:** List of COVID-19 trials COVID-19: coronavirus disease 2019; FDA: food and drug administration; SARS-CoV-2: severe acute respiratory syndrome coronavirus 2; ECMO: extra-corporeal membrane oxygenation; ICU: intensive care unit; RCT: randomized controlled trial; CPAP: continuous positive airway pressure; mRNA: messenger ribonucleic acid; UK: United Kingdom; US: United States; RECOVERY: Randomised Evaluation of COVID-19 Therapy; ACTT-1: Adaptive COVID-19 Treatment Trial-1; ELSO: Extracorporeal Life Support Organization; COVID-PRONE: Prone Positioning for Patients on General Medical Wards With COVID-19; EPIC-HR: Evaluation of Protease Inhibition for Covid-19 in High-Risk Patients; ACT: Anti-Coronavirus Therapies; CPAP: continuous positive airway pressure; RECOVERY-RS: Randomized Evaluation of COVID-19 Therapy–Respiratory Support; REMAP-CAP: Randomized Embedded Multifactorial Adaptive Platform for Community-acquired Pneumonia; BLAZE-1: Blocking Viral Attachment and Cell Entry with SARS-CoV-2 Neutralizing Antibodies; EUA: emergency use authorization; HALT-COVID-19: inHALation of cliclesonide for Treatment of COVID-19

Treatments							
Study/Year	Treatment (Interventions)	Study Design	Stratification Methods	No of Participants (n)	Outcomes	Limitations	Other events/observations
COVID-19 Convalescent Plasma, September 2020 (https://www.covid19treatmentguidelines.nih.gov/therapies/antivirals-including-antibody-products/covid-19-convalescent-plasma/)	Convalescent Plasma	FDA-initiated, national, multicenter, open-label to evaluate the safety of convalescent plasma for patients at high risk of progression to) severe or life-threatening COVID-19	Open-label	20,000	Convalescent plasma was safe and likely to reduce mortality	Convalescent plasma therapy had to be given early in the course of the disease	Subsequent trials found no consistent evidence of benefit
RECOVERY trial, October 2020 [236]	Lopinavir 400 mg plus ritonavir 100 mg by mouth every 12 hours for 10 days or until discharge	Randomized, controlled, open-label	2:1:1 Standard of care alone or usual standard of care plus lopinavir-ritonavir (400 mg and 100 mg, respectively) by mouth for 10 days or until discharge (or one of the other RECOVERY treatment groups: hydroxychloroquine, dexamethasone, or azithromycin	1616	Lopinavir–ritonavir was not effective	Open-label, very few patients on ventilators were enrolled	It is unclear whether the dose of lopinavir-ritonavir we used achieved adequate SARS-CoV-2 inhibitory concentrations in the lungs
Remdesivir-ACTT-1, November 2020 [237]	Remdesivir vs placebo	Double-blind, randomized, placebo-controlled trial	1:1 ratio to receive remdesivir (200 mg loading dose on day 1, followed by 100 mg daily for up to nine additional days) or placebo for up to 10 days.	1062	Remdesivir was superior to placebo in shortening the time to recovery in adults who were hospitalized with COVID-19.	Data on 4.8% of the study were unblinded to provide data to the sponsor; the study had a crossover during this early phase of COVID-19.	The primary outcome of the current trial was changed early in the trial, from a comparison of outcomes on day 15 to a comparison of time to recovery up to day 29.
Coalition I, November 2020 [238]	Hydroxychloroquine, azithromycin, or standard of care	Open-label three group RCT	Randomly assigned in a 1:1:1 ratio to receive standard care, standard care plus hydroxychloroquine, or standard care plus hydroxychloroquine at a dose of 400 mg twice daily plus azithromycin for seven days for mild-to-moderate COVID-19	667	Did not improve clinical status at 15 days	Could not rule out either a substantial benefit or substantial harm.	Other trials did not show benefits for post-exposure prophylaxis in mild COVID-19.
Solidarity trial, January 2021 [239]	Remdesivir, lopinavir/ritonavir combined with interferon-beta, and hydroxychloroquine or chloroquine.	Open-label phase III-IV clinical trial organized by the WHO, Participants were randomly allocated to get one of the 4 therapies	Random assignment to receive one of the 4 treatments	14,304	Hospitalized patients with COVID-19 treated with redeliver had lower death rates and reduced need for oxygen, but no difference in patients already on mechanical ventilation	The trial period had outbreaks due to different strains, delta, and omicron variants. Vaccinations also became more widespread during the trial. Mechanical ventilators were available in resource-limited countries.	WHO suspended the hydroxychloroquine arm of the Solidarity trial in late May 2020
RECOVERY, February 2021 [240]	The usual standard of care alone or the usual standard of care plus oral or intravenous dexamethasone (at a dose of 6 mg once daily) for up to 10 days or until hospital discharge	Randomized, controlled, open-label	2:1 ratio randomization to receive usual care alone or usual standard of care plus oral or intravenous dexamethasone	2104	Lower 28-day mortality among those on invasive mechanical ventilation or oxygen	Open-label, the standard of care varied during the trial period	Patients in the dexamethasone group had a shorter duration of hospitalization than those in the usual care group
INSPIRATION, March 2021 [241]	Intermediate vs. standard prophylactic dose Enoxaparin	RCT, 10 academic centers in Iran	Intermediate dose (enoxaparin, 1 mg/kg daily) vs. standard prophylactic anticoagulation (enoxaparin, 40 mg daily), with modification according to body weight and creatinine clearance. The assigned treatments were planned to be continued until the completion of the 30-day follow-up.	600	No differences in venous or arterial thrombus, treatment with ECMO, or mortality within 30 days	Open-label enrolled ICU patients but not severely ill patients requiring ECMO, lack of systematic screening for venous thromboembolism, only four patients were >120kg	increased risk of bleeding (2.5%) in the intermediate dose group, which did not meet non-inferiority criteria; severe thrombocytopenia only occurred in the intermediate dose group.
COVACTA, May 2021 [242]	Standard of care alone versus usual standard of care plus tocilizumab at a dose of 400 mg–800 mg	Open-label	21,550 patients enrolled in the RECOVERY trial were included in the assessment of tocilizumab	4116	Tocilizumab improved survival	Duration of hospitalization beyond 28 days was not recorded	Of patients in the tocilizumab group, 16% did not receive this treatment, and the reasons for this were not recorded
Heparin, August 2021 [243]	Therapeutic-dose anticoagulation with heparin vs. pharmaco- logic thromboprophylaxis	Open-label RCT, critically ill patients with severe COVID-19	1:1 randomization to therapeutic-dose anticoagulation with heparin vs. pharmaco- logic thromboprophylaxis in accordance with local usual care	1098	No differences in survival to hospital discharge or a greater number of days free of cardiovascular or respiratory organ support	Open-label, most patients were from the UK, practice guidelines changed during the trial period, and the majority received intermediate-dose anticoagulation in the control group.	The incidence of major bleeding was 3.8% in the treatment group.
ECMO, September 2021 [244]	Outcomes after ECMO in COVID-19	Analysis of the ELSO Registry and COVID-19 for between-group comparison of ECMO-supported patients with COVID-19	Retrospective analysis of ECMO registries	4812	Mortality for ECMO-supported patients with COVID-19 worsened worldwide throughout the pandemic, and the duration of ECMO support increased.	Not an RCT, did not give insight about which patients need ECMO support	Mortality was 38-59%
Remdesivir plus standard of care-DisCoVeRy, September 2021 [245]	Remdesivir plus standard of care	Phase-3, open-label, adaptive, multicentre, randomized, controlled trial conducted in 48 sites in Europe (France, Belgium, Austria, Portugal, Luxembourg) between March 22, 2020, and January 21, 2021,	1:1 randomization, Remdesivir was administered intravenously at a loading dose of 200 mg on day 1, followed by 100 mg, for a total duration of 10 days. Its cessation was allowed after five days if the participant was discharged from the hospital.	857	No clinical benefit was observed from the use of remdesivir in symptomatic patients for more than seven days and required oxygen support.	Open-label, several treatments were concomitantly evaluated during the trial period; no viral load assessment was available at any time point.	Among the subset of participants without mechanical ventilation or ECMO at randomization, remdesivir significantly delayed the need for new mechanical ventilation or ECMO, or death, consistent with what was reported in ACTT-1.9.
Aspirin-RECOVERY, November 2021 [246]	Aspirin	Open-label RCT, 177 hospitals in the UK, two hospitals in Indonesia, and two hospitals in Nepal, November 1, 2020, and March 21, 2021	1:1 ratio to either the usual standard of care plus 150 mg aspirin once per day until discharge or the usual standard of care alone	14892	No reduction in 28-day mortality, mechanical ventilation, or death, a small increase of one median day of being discharged alive.	Open-label, only hospitalized patients were studied, radiological information was not collected,	The effects of aspirin were similar to other trials of patients with cardiovascular disease.
COVID-PRONE, January 2022 [247]	Proning in patients requiring upto 50% fraction of inspired oxygen but not critically ill	Randomized 1:1 to prone positioning	Patients were randomized 1:1 to prone positioning (that is, instructing a patient to lie on their stomach while they are in bed) or standard of care	570	Prone positioning did not improve outcomes in hospitalized hypoxemic patients	Adherence to prone positioning was poor, despite multiple efforts to increase it; time spent prone was self-reported and thus at risk of recall bias,	The median total time spent in prone position up to the first 72 hours was 6 hours
CPAP-RECOVERY-RS, January 2022 [248]	CPAP vs. conventional oxygen	Parallel group, adaptive, randomized clinical trial from April 6, 2020, and May 3, 2021, across 48 acute care hospitals in the UK and Jersey	1:1 randomization	1273	The initial strategy of CPAP significantly reduced the risk of tracheal intubation or mortality compared with conventional oxygen therapy.	Cross over occurred	Treatment crossover occurred in 17.1% of participants.
Early treatment with Ivermectin, March 2022 [249]	Ivermectin	Double-blind, randomized, placebo-controlled, adaptive platform trial, 12 public health clinics in Brazil.	Patients were randomized to receive ivermectin (679 patients), placebo (679), or another intervention (2157). ivermectin (400 μg per kilogram of body weight) once daily for 3 days or placebo.	3515	Did not result in a lower incidence of medical admission to a hospital due to the progression of disease among outpatients with an early diagnosis of COVID-19.	Enrolled patients only in Brazil; although 3515 patients were enrolled, ivermectin vs. placebo was compared only in 679 patients.	There was no evidence of a treatment effect with ivermectin as compared with placebo in subgroups defined according to patient age, body-mass index, status of having cardiovascular disease or lung disease, sex, smoking status, or time since symptom onset.
Colchicine and Rixaroxaban-ACT Trial, October 2022 [250]	Colchicine and Rivaroxaban + Aspirin	Open-label RCT, 62 clinical centers in 11 countries between Oct 2, 2020, and Feb 10, 2022	colchicine 1·2 mg followed by 0·6 mg 2 h later within 72 hrs of hospitalization and then 0·6 mg twice daily for 28 days versus usual care; and in a second (1:1) randomization, to the combination of rivaroxaban 2·5 mg twice daily plus aspirin 100 mg once daily for 28 days versus usual care	2749	No prevention of disease progression or death	Open-label, the trial was done over 18 months and, different therapies were used, different strains emerged, vaccinations increased as the trial progressed	56 to 65% of patients were unvaccinated.
Nirmatrelvir and Ritonavir EPIC-HR, February 2022 [251]	300 mg of nirmatrelvir plus 100 mg of ritonavir vs placebo	Phase 2-3 double-blind, randomized, controlled trial	1:1 ratio to receive either 300 mg of nirmatrelvir plus 100 mg of ritonavir for 5 days	2246	The risk of progression to severe COVID-19 was 89% lower than the risk with a placebo	The median age was only 45 years, and the predominantly white population	The trial was restricted to unvaccinated persons
Long-term cardiovascular outcomes of COVID-19, March 2022 [252]	Prospective cohort	Data was reported from the US Department of Veterans Affairs National Healthcare databases	The healthcare database was used to build a cohort and then followed longitudinally to estimate the risks and 12-month burdens of pre-specified incident cardiovascular outcomes	153,760	risk and 12-month burden of ischemic and non-ischemic heart disease, dysrhythmias increased post COVID-19	Majority were males	Increased risk of myocarditis and pericarditis reported in this study is significant in people who were not vaccinated and is evident regardless of vaccination status.
Long-term (180-Day) Outcomes in Critically Ill Patients With COVID-19, REMAP-CAP, December 2022 [253]	Follow-up study	Randomized to receive one or more interventions within six treatment domains: immune modulators, convalescent plasma, antiplatelet therapy (n=1557), anticoagulation, antivirals, and corticosteroids		4869	IL-6 receptor antagonist had a greater than 99.9% probability of improved 180-day mortality	Open-label, not all centers collected quality of life and disability scores	One in three patients had at least moderate disability that persisted through six months
Nirmatrelvir–ritonavir, February 2023 [254]	Nirmatrelvir–ritonavir for reducing hospital admissions and mortality from COVID-19	Population-based cohort study	Population-based follow-up study	177,545	Significantly reduced odds of hospital admission and death from COVID-19,	Study tracked prescriptions, could not confirm adherence	Number needed to treat= 62.
Prevention							
Phase 3 of BLAZE-1 trial, Bamlanivimab plus Etesevimab, July 2021 [255]	2800 mg of Bamlanivimab and 2800 mg of Etesevimab within three days in mild or moderate COVID-19	Double-blind, placebo-controlled, RCT, single-dose trial conducted in the US	Ambulatory patients with mild or moderate COVID-19, who were at high risk for progression to severe disease were randomized in a 1:1 ratio to receive a single intravenous infusion of either a neutralizing monoclonal-antibody combination agent (2800 mg of bamlanivimab and 2800 mg of etesevimab, administered together) or placebo within 3 days after a laboratory diagnosis. The primary outcome was the overall clinical status of the patients, defined as COVID-19–related hospitalization or death from any cause by day 29.	1035	Bamlanivimab plus etesevimab resulted in more rapid resolution of symptoms within four days after the initiation of treatment, less hospitalization and death compared to placebo, and accelerated decline in the SARS-CoV-2 viral load.	12.6% were White people, conducted only in the US with very few adolescents, emergency use authorization for bamlanivimab plus etesevimab is for administration within 10 days but was administered within three days in the study	On April 16, 2021, the FDA revoked the EUA for the investigational monoclonal antibody therapy bamlanivimab to be used alone
REGEN-COV, September 2021 [256]	Combination of the monoclonal antibodies casirivimab and imdevimab, REGEN-COV 2400-mg vs placebo	Phase 3, randomized controlled trial	1:1:1 ratio to receive intravenous REGEN-COV at a dose of 2400 mg (1200 mg each of casirivimab and im-devimab) or 8000 mg (4000 mg of each anti-body) or intravenous placebo	2696	REGEN-COV in outpatients with high-risk factors for severe COVID-19 dramatically reduced the risk of hospitalization, symptom duration and all-cause death by 70.4%	Efficacy against other strains was not studied.	FDA has revised the EUAs for bamlanivimab/etesevimab and casirivimab/imdevimab (REGEN-COV) and are currently not authorized for use due to inactivity against the Omicron variant
Vaccines							
Safety and Efficacy of the BNT162b2 mRNA COVID-19 Vaccine (Pfizer), December 2020 [257]	BNT162b2 mRNA vaccine vs. placebo	Randomized placebo-controlled, observer-blinded	1:1 ratio to receive two doses, 21 days apart, of either placebo or the BNT162b2 vaccine	43,548	The BNT162b2 two-dose vaccine provided 95% protection against COVID-19 in individuals aged 16 years and above. Its safety profile during a median follow-up of 2 months was comparable to other viral vaccines.	The endpoints in protocols for different vaccines are different, and it is difficult to compare efficacy across different vaccine groups	Further studies showed that the bivalent vaccine was 58.7% effective against hospitalization compared to 25% for the monovalent one that preceded it
AstraZeneca COVID vaccine AZD1222 against SARS-CoV-2, January 2021 [258]	ChAdOx1 nCoV-19 vaccine (AZD1222)	Randomized, blinded, controlled trials were done across three countries: COV001 (phase 1/2; UK), COV002 (phase 2/3; UK), COV003 (phase 3; Brazil), and COV005 (phase 1/2; South Africa) between April 23 and November 4, 2020	Two standard doses vs. one low dose followed by a standard dose	23,848	The vaccine was well tolerated and efficacy against COVID-19 was 80%.	The endpoints in protocols for different vaccines are different, and it is difficult to compare efficacy across different vaccine groups.	
Efficacy and Safety of the mRNA-1273 Vaccine (Moderna), February 2021 [259]	mRNA-1273 vaccine vs. placebo	Phase-3 randomized, observer-blinded, placebo-controlled trial	1:1 ratio to receive two intra- muscular injections of mRNA-1273 (100 μg) or a placebo 28 days apart.	30,420	The vaccine conferred 94.1% efficacy against COVID-19, including severe disease.	The endpoints in protocols for different vaccines are different, and it is difficult to compare efficacy across different vaccine groups.	Further studies showed effectiveness against the XBB strains varied by age: in ages 18-49 years, it was 49% against the XBB strains versus 52% against the BA.5 viruses; in ages 50-64 years, it was 40% compared to 43% for BA.5; and in people aged 65 and older, 43%, compared to 37% for the BA.5 viruses.
ENSEMBLE trial-Johnson and Johnson vaccine, April 2021 [260]	Ad26.COV2.S vaccine against COVID-19	Randomized, double-blinded, placebo-controlled trial	1:1 ratio to receive a single dose of Ad26.COV2.S (5×1010 viral particles) or placebo.	39,321	Safety appeared to be similar to that in other phase-3 trials of COVID-19 vaccines.	The endpoints in protocols for different vaccines are different, and it is difficult to compare efficacy across different vaccine groups.	
Efficacy and Safety of NVX-CoV2373 (Novavax), February 2022 [261]	NVX-CoV2373 vaccine vs. placebo	Phase-3, randomized, observer-blinded, placebo-controlled trial at 113 clinical sites	2:1 ratio to receive two doses of NVX-CoV2373 or placebo 21 days apart	29,949	Two doses of NVX-CoV2373 were safe	The endpoints in protocols for different vaccines are different, and it is difficult to compare efficacy across different vaccine groups.	Had a blinded crossover approximately three to four months after the first vaccination series to allow all trial participants to receive NVX-CoV2373 after vaccine efficacy and required safety had been established and reviewed
COVID and risk of incident diabetes, December 2022 [262]	COVID vaccine	Self-controlled crossover observational cohort, Cedars-Sinai Health System in Los Angeles, California, from March 2020 to June 2022		23,709	The risk of type 2 diabetes after COVID infection was higher in unvaccinated individuals compared to vaccinated individuals.		
Ongoing trials							
COVERAGE, A Early Treatment of Vulnerable Individuals With Non-Severe SARS-CoV-2 Infection (https://clinicaltrials.gov/study/NCT04920838)	Early treatment of vulnerable individuals with non-severe SARS-CoV-2						
Solidarity PLUS Solidarity Finland Plus Long-COVID (https://clinicaltrials.gov/study/NCT05220280)	Enroll hospitalized patients to test artesunate, imatinib, and infliximab						

Importance of Ongoing Trials to Fight COVID-19

There is a need for further research in various fields for the effective management of high-risk patients and complications. The emergence of new virus variants hinders the usefulness of existing drugs. COVID-19 in immunosuppressed states, malignancies, inherited or acquired immunodeficiencies, and solid organ transplants have a particularly devastating course and need further therapies to improve outcomes. In the future, certain COVID-19 study platforms may remain and evolve to address other infections. Long COVID is presently a major cause of disability. This complex post-COVID-19 entity is more severe than other post-viral syndromes. Some antifibrotic or anti-inflammatory drugs are currently being studied to prevent Long COVID [263]. While countless studies have improved our understanding of COVID-19, there remain many unanswered, unknown variables, and we are optimistic that they will be answered in future trials.

Medications

**Figure 8:**
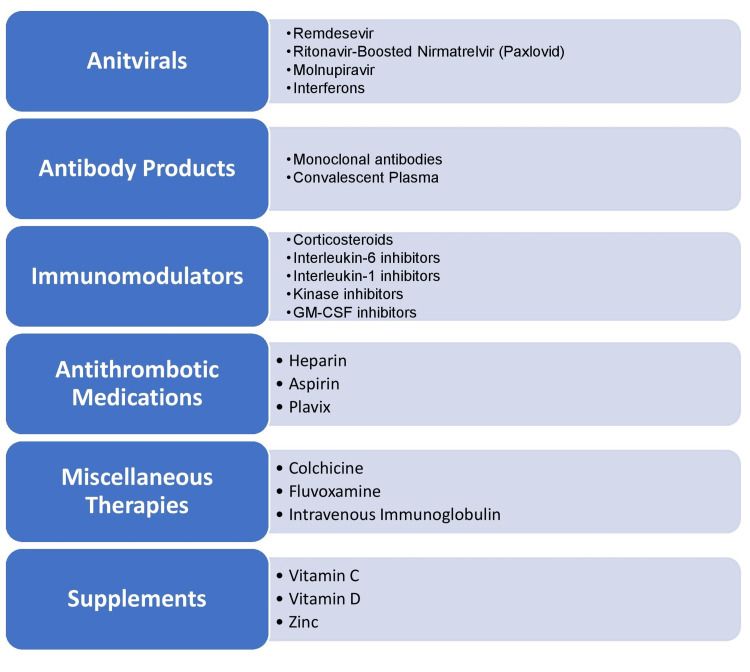
. Medications used to date in the treatment of coronavirus disease 2019 (COVID-19) infection. GM-CSF: granulocyte-macrophage colony-stimulating factor

Here, we discuss the various treatment options for COVID-19 infection, emphasizing the management of patients with an immunocompromised state (transplant, cancer), HIV, pregnancy, and co-infection with influenza. Since the beginning of the pandemic, multiple medications have been utilized in treating patients diagnosed with COVID-19 infection, as listed in Figure 8.

Antivirals

The FDA has approved the use of remdesivir and issued an emergency use authorization (EUA) for Paxlovid and Molnupiravir in patients who test positive for COVID-19 with mild to moderate symptoms (not requiring supplemental oxygen or an increase in home oxygen, non-hospitalized patients).

Remdesevir is a nucleotide analog with an inhibitory function against SARS-CoV-2 [264]. During the early phase of the pandemic, a study by Beigel et al. [265] showed that remdesivir shortened the time to recovery in patients admitted with severe COVID-19 receiving supplemental oxygen therapy, which was later shown to be ineffective in patients symptomatic for over seven days and requiring supplemental oxygen. WHO recommended against using remdesivir in hospitalized patients citing no benefits in clinical outcomes. A trial in 2022 showed that three-day outpatient treatment with intravenous remdesivir resulted in an 87% reduction in the risk of hospitalizations or death in patients with symptoms for less than seven days [266].

Ritonavir-boosted nirmatrelvir (Paxlovid) is an oral SARS-CoV-2 Mpro inhibitor [267], packaged with ritonavir (cytochrome P450 (CYP) 3A4 inhibitor and a boosting agent used for HIV protease inhibitors) to increase the concentration of nirmatrelvir to the desired therapeutic range [268]. Paxlovid can be used in high-risk symptomatic COVID-19, non-hospitalized patients resulting in a lower risk of progression to severe COVID-19. Caution must be used to assess the safety of Paxlovid (ritonavir component) due to concern for significant drug-drug interactions. As of May 25, 2023, Paxlovid is the first oral antiviral drug approved by the FDA for use in high-risk patients for progression to severe COVID-19, including hospitalization or death.

Molnupiravir is an oral ribonucleoside, a prodrug of beta-D-N4-hydroxycytidine (NHC) with antiviral activity against SARS-CoV-2. Molnupiravir is approved for use in non-hospitalized patients at high risk for disease progression, starting within five days of the onset of symptoms [269]. A meta-analysis [270] showed that patients receiving molnupiravir had reduced all-cause mortality and risk of hospitalizations and improved proportions of patients testing negative by day 5 for viral RNA. It is recommended to be used only when remdesivir and Paxlovid are unavailable.

Interferons are cytokines with antiviral properties. Studies conducted during the early phases of the pandemic administered interferons in addition to other drugs for COVID-19, such as hydroxychloroquine, lopinavir/ritonavir, and ribavirin [271-273]. RCTs did not show the clinical benefit of adding interferon to remdesivir, with likely worse outcomes in patients needing high-flow oxygen therapy [274] along with no reduction in length of stay, need for mechanical ventilation, or mortality [275].

Antibody products

Monoclonal antibodies targeting the SARS-CoV-2 spike protein have shown some clinical benefits in treating COVID-19 infection. Four neutralizing monoclonal antibodies received EUA previously include bamlanivimab with etesevimab, bebtelovimab, casirivimab with imdevimab, and sotrovimab. Since the emergence and dominance of Omicron and the unknown efficacy of these drugs against this variant, they are currently not approved for use.

Bamlanivimab with etesevimab neutralizes antibodies obtained from the convalescent plasma of patients who recovered from COVID-19 infection in North America and China, respectively. Etesevimab binds to a different epitope than bamlanivimab and is shown to neutralize variants with resistant mutations on the epitope bound to bamlanivimab [276]. Results from phase 3 of BLAZE-1 suggested an 87% reduction in COVID-19-related hospitalizations or death in patients in the treatment group as compared to the patients receiving placebo [277].

Bebtelovimab is a potent anti-spike neutralizing antibody with activity against all known SARS-CoV-2 variants of concern in vitro [278]. In the ongoing phase-2 BLAZE-4 trial, non-hospitalized patients with mild to moderate COVID-19 treated with bebtelovimab alone or combined with bamlanivimab and etesevimab showed significant reductions in viral load and reduced time to sustained symptom resolution.

Casirivimab with imdevimab (REGEN-COV) is an antibody combination that significantly reduced COVID-19-related hospitalizations or all-cause mortality compared to a placebo.

Sotrovimab is also a potent anti-spike neutralizing antibody targeting a region of the SARS-CoV-2 epitope not competing with the binding of the ACE-2 [279]. In the COMET-ICE study, non-hospitalized patients with mild to moderate COVID-19 were randomized to either receive sotrovimab or placebo. All-cause hospitalization and death were significantly reduced in the patients receiving sotrovimab [279].

Tixagevimab and cilgavimab (Evusheld) are long-acting monoclonal antibodies used as pre-exposure prophylaxis for COVID-19 in patients ineligible for vaccinations due to severe allergy or immunocompromized states. Preliminary data from the PROVENT (Phase III Double-blind, Placebo-controlled Study of AZD7442 for Pre-exposure Prophylaxis of COVID-19 in Adult) trial suggests a significant reduction in the risk of experiencing symptomatic COVID-19 infection when patients were treated with tixagevimab and cilgavimab compared to placebo [280].

COVID-19 convalescent plasma (CCP) obtained from the plasma of patients recovered from COVID-19 infection has received the US FDA EUA for immunocompromised patients in hospitalized or non-hospitalized settings. Several RCTs [281-283] evaluating the use of high-titer CCP in immunocompetent patients hospitalized with COVID-19 showed no benefit, resulting in the early discontinuation of these studies. Although there is limited evidence to support its use in the immunocompromised [283,284], a recent systematic review and meta-analysis by Senefeld et al. suggested a mortality benefit in immunocompromised patients with COVID-19 [285].

Immunomodulators

Corticosteroids remain the standard of care for patients with severe COVID-19 infection requiring hospitalization and oxygen therapy. They help diminish the systemic inflammatory response seen in COVID-19 infection leading to lung injury and multi-organ failure. They have been shown to improve clinical outcomes and reduce mortality in patients hospitalized with COVID-19, requiring supplemental oxygen therapy [286,287]. In the RECOVERY trial, hospitalized patients with COVID-19 receiving respiratory support ranging from supplemental oxygen to mechanical ventilation and receiving oral or intravenous dexamethasone for up to 10 days had lower mortality than those receiving usual care without any respiratory support. In a study by Crothers et al., the early use (within 48 hours) of dexamethasone in hospitalized patients with COVID-19 on no respiratory support or only nasal cannula oxygen did not provide mortality benefit with concern for potential harm [288]. Inhaled budesonide and ciclesonide used in the outpatient setting in COVID-19 patients did not consistently alleviate symptoms of COVID-19 infection and did not affect the rate of hospitalization or death [289-292].

IL-6 inhibitors include anti-IL-6 receptor monoclonal antibodies (sarilumab, tocilizumab) and anti-IL-6 monoclonal antibodies (siltuximab). IL-6 are proinflammatory cytokines produced by the fibroblasts, lymphocytes, and monocytes. COVID-19 infection is associated with a surge in cytokine releases, such as CRP, D-dimer, ferritin, and IL-6, which is responsible for widespread inflammation, particularly respiratory failure [293,294].

Tocilizumab is a recombinant anti-IL-6 receptor monoclonal antibody approved for use in patients with rheumatologic disorders, chimerin antigen receptor T cell therapy-induced cytokine release syndrome (CRS), and select hospitalized patients with COVID-19 infection. In both RECOVERY and REMAP-CAP trials, survival benefit was seen in patients experiencing rapid respiratory failure and elevated CRP levels receiving tocilizumab compared to the standard of care [295,296]. However, in a double-blind, RCT assessing the use of tocilizumab and remdesivir to placebo and remdesivir, tocilizumab did not shorten the time to hospital discharge and was not associated with reduced mortality.

Sarilumab is also a recombinant anti-IL-6 receptor monoclonal antibody approved for managing rheumatoid arthritis. In a phase-3 trial, sarilumab failed to show mortality benefit in hospitalized patients with COVID-19 requiring supplemental oxygen therapy [297]. In the REMAP-CAP trial, similar to tocilizumab, sarilumab showed a higher likelihood of survival during hospitalization and greater organ support-free days [296]. Sarilumab use is recommended only during the non-availability or inability of tocilizumab use.

Siltuximab is a recombinant IL-6 monoclonal antibody that inhibits IL-6 signaling by preventing the binding of IL-6 to both membrane-bound and soluble IL-6 receptors. They are approved for use in Castleman disease. Due to the limited availability of data on the efficacy of siltuximab, they are not recommended for use in patients with COVID-19 infection.

Janus Kinase (JAK) Inhibitors interfere with the phosphorylation of signal transducer and activator of transcription (STAT) proteins, hence stunting cellular signaling, growth, and survival [298]. They are involved in preventing immune activation and inflammation by the release of proinflammatory cytokines and by inhibiting the phosphorylation of the signal transduction proteins, thus showing a role to play in the treatment of COVID-19 [299]. Multiple JAK inhibitors, including baricitinib, tofacitinib, and ruxolitinib, have been studied to treat COVID-19; however, only baricitinib and tofacitinib are recommended for use.

Baricitinib is a selective inhibitor of JAK1 and JAK2. It is thought to impart its antiviral effects by preventing the entry of SARS-CoV-2 into the lung alveolar epithelial cells [300]. Baricitinib is FDA-approved for use in rheumatoid arthritis and hospitalized COVID-19 patients requiring oxygen therapy, including patients on non-invasive ventilation (NIV), mechanical ventilation, or ECMO. In both RECOVERY and COV-BARRIER trials [301,302], the use of baricitinib showed survival benefits in hospitalized patients receiving supplemental oxygen via a high-flow device or non-invasive ventilation, with additional benefits noted in patients on mechanical ventilation in the latter trial. In the ACTT-2 and ACTT-4, baricitinib was shown to be potentially beneficial; however, neither trial evaluated the benefit of the use of dexamethasone [303,304].

Tofacitinib is a selective inhibitor of JAK1 and JAK3, with some activity against JAK2. It is FDA-approved for rheumatoid arthritis, juvenile idiopathic arthritis, rheumatoid arthritis, and ulcerative colitis. In a double-blind, placebo-controlled randomized trial [305], treatment with tofacitinib, as compared to placebo, carried a lower risk of mortality or respiratory failure in the hospitalized patients with COVID-19, a majority of whom were treated with systemic glucocorticoids.

Bruton’s tyrosine kinase (BTK) Inhibitors are B-cell and cytokine receptor signaling molecules approved by the FDA to treat various B-cell malignancies. The three drugs in this class include acalabrutinib, ibrutinib, and zanubrutinib.

Heparin: COVID-19 has been associated with an increased incidence of thrombo-embolic events resulting in increased mortality [294]. Therefore, all individuals hospitalized with COVID-19 should receive thrombus prophylaxis with low-molecular-weight heparin or unfractionated heparin. Depending on disease severity, recommendations regarding doses have changed during the pandemic. It is important to consider individual risk assessment when deciding on these patients' anticoagulation [306]. Based on the analysis of recent trials (ACTION, RAPID, HEP-COVID) in hospitalized non-ICU patients, a therapeutic dose of anticoagulation has been associated with better outcomes [307-309]. So current guidelines suggest using a therapeutic dose of anticoagulation for prophylaxis if not contraindicated otherwise. Dose adjustment may be needed based on individual risk factors. In ICU patients, a prophylactic dose of anticoagulation is preferred. COVID-19 patients already on therapeutic anticoagulation should not reduce the dose unless there is a contraindication. Anticoagulation continuation is not recommended on discharge in either group.

Aspirin: Aspirin or other antiplatelets (Plavix/Ticagrelor) are not recommended for treating patients with COVID-19 in either inpatient or outpatient based on major trials, including RECOVERY, ACTIV-4A, and ACTIV-4B. Individuals already on antiplatelet therapy for other indications may continue it if not contraindicated otherwise [310].

Colchicine: Oral colchicine did not significantly reduce mortality based on RCTs, including over 4000 patients. The use of colchicine in the treatment of COVID-19 is not recommended [311].

Fluvoxamine is an antidepressant medication, and its use for treating COVID-19 has not been recommended. An RCT in patients with mild to moderate COVID-19 did not show significant benefit with fluvoxamine [312]. Vitamin C is not associated with a reduction in the incidence or severity of COVID-19. Vitamin D has no benefit in the treatment of COVID-19. Zinc may decrease the duration of symptoms in mild COVID-19, but there is not enough evidence regarding improving outcomes [313]. Intranasal use of zinc may result in hyposmia or anosmia.

Management of respiratory failure in COVID-19

All patients with COVID-19 should be monitored closely for any signs of respiratory decompensation using pulse oximetry (SpO2) as progressive respiratory decline can lead to ARDS and eventually multi-organ failure and death.

Monitoring Oxygenation

The target SpO2 to be maintained in patients with COVID-19 remains largely unclear. In patients with hypoxemia, the goal of providing supplemental oxygen therapy should be to maintain SpO2 between 92% and 96% [314]. A systematic review and meta-analysis of 25 RCTs showed that liberal oxygen supplementation in patients with respiratory failure without COVID-19 to maintain SpO2 around 94-96% was associated with increased mortality [315]. In a study by Barrot et al., conservative oxygen therapy to maintain SpO2 between 88% and 92% in patients with ARDS without COVID-19 did not show increased survival at 28 days [316]. However, caution must be exercised while interpreting SpO2 in patients with dark skin, as occult hypoxemia may go undetected in these patients by pulse oximetry [317].

Acute Hypoxemic Respiratory Failure

COVID-19 is frequently associated with acute respiratory failure with varying degrees of hypoxemia. Conventional oxygen supplementation can be inadequate to meet the oxygen demands in these patients prompting escalation of respiratory support with a variety of modalities, including high-flow nasal cannula (HFNC), non-invasive ventilation (NIV), intubation and mechanical ventilation (MV), or extracorporeal membrane oxygenation (ECMO).

HFNC oxygen therapy: HFNC therapy involves delivery, to the nose, of a blend of humidified and heated oxygen and air at high flow rates [318]. In a study by Frat et al., 310 patients with acute hypoxemic respiratory failure were randomly assigned to HFNC, oxygen therapy via a face mask or NIV. The primary outcome of the intubation rate at day 28 did not differ amongst any of the three treatment modalities; however, patients receiving HFNC therapy experienced a significantly lower risk of death at 90 days [319]. Subsequently, a systematic review and meta-analysis of eight trials totaling 1084 patients showed that intubation and ICU mortality rates were lower in patients treated with HFNC compared to conventional oxygen therapy and NIV [320].

Since the beginning of the pandemic, multiple studies have reported on the utility of HFNC in managing respiratory failure with COVID-19. A RCT evaluating 220 patients with severe COVID-19 found that the rate of intubation and time to clinical improvement was significantly lower in patients treated with HFNC oxygen therapy compared to conventional oxygen therapy [321].

NIV: Bilevel positive airway pressure (BiPAP) and continuous positive airway pressure (CPAP) constitute the NIV utilized to manage respiratory failure from COVID-19. Determination of the ideal timing of initiation of this modality, in comparison with HFNC, in COVID-19 patients with progressive respiratory failure remains unclear to date. Furthermore, NIV is an aerosol-generating procedure and may be associated with an increased risk of nosocomial transmission of SARS-CoV-2 [322].

In the Helmet Noninvasive Ventilation Versus High-Flow Oxygen Therapy in Acute Hypoxemic Respiratory Failure (HENIVOT) study involving 109 patients with COVID-19-associated moderate to severe respiratory failure, NIV via a helmet device, as compared to HFNC, did not lead to a significant difference in respiratory support-free days however was associated with lower rates of endotracheal intubation which was statistically significant [323].

RECOVERY-RS was a large trial evaluating 1273 patients randomized to use either CPAP or HFNC for acute respiratory failure with hypoxemia due to COVID-19 in comparison to conventional oxygen therapy. The primary outcomes of endotracheal intubation or 30-day mortality were significantly lower in patients receiving CPAP compared to conventional oxygen therapy, thus favoring CPAP as the initial strategy for managing respiratory failure in COVID-19 [324]. The results of this study need careful interpretation as it was terminated early and hence underpowered, coupled with the crossover between the groups.

Prone positioning: In patients with moderate to severe ARDS needing mechanical ventilation, early prone positioning (PP) has been associated with improved oxygenation and outcomes, as seen in the Proning Severe ARDS Patients (PROSEVA) RCT [325]. PP has improved oxygenation in COVID-19 patients needing supplemental oxygen or NIV, with some reports of a lower need for intubation [326,327].

Awake Prone Positioning Meta-Trial Group is the largest trial, including 1126 patients randomized to either PP along with HFNC or standard care (HFNC alone) [328]. Patients had a lower incidence of intubation by day 28 in the PP group vs the standard care group; however, no difference was seen in the 28-day mortality between the groups. The median duration of awake PP was about 5 hours, and longer durations of awake PP resulted in treatment success by day 28. A systematic review and meta-analysis by Munshi et al. of 8 RCTs totaling 2129 patients with ARDS evaluated PP use [329]. Patients in the PP group for >12 hours had lower mortality than the supine position group, and PP was noted to improve oxygenation in all trials.

MV: In COVID-19 patients with progressively worsening acute hypoxemic respiratory failure, MV should be offered when all other non-invasive modalities of improving oxygen fail. Akin to ventilator management in ARDS, positive end-expiratory pressure (PEEP) plays a significant role in COVID-19 patients by improving oxygenation, preventing alveolar collapse, and reducing atelectotrauma, thus minimizing ventilator-induced lung injury.

In the pre-pandemic systematic review and meta-analysis of three trials, Briel et al. compared the management of ARDS with high vs low PEEP. The results suggested that higher levels of PEEP were associated with lower ICU and in-hospital mortality rates [330]. In COVID-19 patients with ARDS (CARDS), despite the moderate to severe ARDS, lung compliance can vary dramatically from normal to low compliance, as typically seen in non-COVID-19 etiologies. In a case series by Tsolaki et al., applying higher PEEP (>10 cm H2O) in CARDS with normal lung compliance was detrimental as it resulted in a significant increase in pleural pressure leading to a decline in venous return leading to hemodynamic compromise. Thus, individualized ventilator management must be formulated based on the patient’s respiratory and hemodynamic status to ensure the best patient outcomes [331].

ECMO: ECMO is a type of cardiopulmonary life support involving blood from the vascular system, pumping it through the body's exterior, and then reinfusing it into circulation. Hemoglobin fully saturates with oxygen outside the body, and CO2 is expelled. The rate of countercurrent gas flow through the oxygenator determines oxygenation, and the amount of CO2 eliminated may be regulated by changing that rate [332]. Indications for ECMO use can be cardiac failure, respiratory failure, or a combination of both [333].

ECMO has been utilized with inconclusive results regarding respiratory outcomes and mortality in patients with CARDS and worsening hypoxemia refractory to other modalities. [329,334,335] In a pre-pandemic study, ECMO use in ARDS did not result in lower 60-day mortality [335]. In a retrospective analysis of 4812 patients treated with ECMO for CARDS, a higher 90-day mortality was noted [244]. Given the lack of controlled trials evaluating ECMO use in patients with CARDS, their benefits remain unproven at this time.

Vaccinations

Immunizations (or vaccinations) are one of the most effective preventive health measures. It is a safe, simple, and effective way to protect populations against harmful diseases.

**Figure 9:**
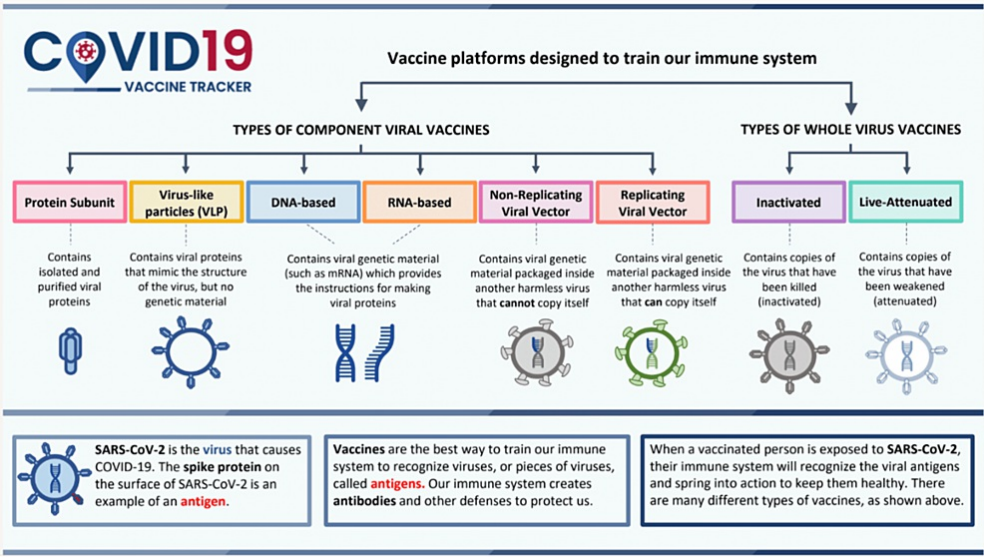
Different types of vaccine platforms DNA: deoxyribonucleic acid; RNA: ribonucleic acid; SARS-CoV-2: severe acute respiratory syndrome-coronavirus-2 Image Source: COVID-19 Vaccine Tracker (https://covid19.trackvaccines.org/vaccine-types/)

There are two categories of COVID-19 vaccines: component viral vaccines and whole virus vaccines (Figure 9). Component viral vaccines consist of isolated and purified viral protein subunits, virus-like particles (VLP) that mimic the structure of the virus without the genetic materials, are DNA-based and RNA-based, and replicating and non-replicating viral vectors. Whole virus vaccines, on the other hand, are inactivated (contain killed viral copies) and live-attenuated (contain weakened viral copies).

The WHO has granted EUL for 11 vaccines, as listed in Table 3. Additionally, about 50 vaccines approved worldwide in at least one country are listed in Table 4.

**Table 3:** COVID-19 vaccines approved by the WHO under emergency use listing SARS-CoV-2: severe acute respiratory syndrome coronavirus 2; WHO: World Health Organization, Covid 19: Coronavirus disease 2019, mRNA: messenger ribonucleic acid

Vaccine	Vaccine Type	Vaccine Platform	Approved Countries	No of Trials	WHO EUL recommendation	Age indication	Shelf life	EUL Holder	Approval Source	Trials Link
COVOVAX	SARS-CoV-2 rS Protein Nanoparticle [Recombinant]	Protein subunit	6	7	17 December 2021	12 years and older	9 months Storage temperature: 2°C to 8°C	Serum Institute of India Pvt. Ltd. (SIIPL)	COVOVAX | WHO - Prequalification of Medical Products (IVDs, Medicines, Vaccines, and Immunization Devices, Vector Control)	Serum Institute of India: COVOVAX (Novavax formulation) – COVID-19 Vaccine Tracker
NOVAVAX	SARS-CoV-2 rS [Recombinant, adjuvanted]	Protein subunit	40	22	20 December 2021	12 years and older	Nine months, storage temperature: 2°C to 8°C	Serum Institute of India Pvt. Ltd. (SIIPL)	NUVAXOVID | WHO - Prequalification of Medical Products (IVDs, Medicines, Vaccines, and Immunization Devices, Vector Control)	Novavax: Nuvaxovid – COVID19 Vaccine Tracker
MODERNA	COVID-19 mRNA Vaccine (nucleoside modified)	RNA	88	70	30 April 2021	6 years and older	Nine months, storage temperature: -20°C ± 5°C	Moderna Biotech	COVID-19 mRNA Vaccine (nucleoside modified)	Moderna: Spikevax – COVID19 Vaccine Tracker
Pfizer/BioNTech	COVID-19 mRNA vaccine (nucleoside modified)	RNA	149	100	31 December 2020	6 months and older	Nine months, storage temperature: - 90°C to 60°C	BioNTech Manufacturing GmbH	WHO recommendation BioNtech Tozinameran – COVID-19 mRNA vaccine (nucleoside modified) – COMIRNATY	Pfizer/BioNTech: Comirnaty – COVID-19 Vaccine Tracker
CanSino	Ad5-nCoV-S [Recombinant]	Non-Replicating Viral Vector	10	14	19 May 2022	18 to 59 years of age	12 months, storage temperature: 2°C to 8°C	CanSino Biologics Inc.	CONVIDECIA | WHO - Prequalification of Medical Products (IVDs, Medicines, Vaccines, Immunization Devices, Vector Control)	CanSino: Convidecia – COVID-19 Vaccine Tracker
Janssen	Ad26.COV2-S [recombinant]	Non-Replicating Viral Vector	113	26	12 March 2021	18 years and older	24 months, storage temperature: -25°C to -15°C	Janssen–Cilag International NV	COVID-19 Vaccine (Ad26.COV2-S [recombinant])	Janssen (Johnson & Johnson): Jcovden – COVID19 Vaccine Tracker
Oxford/AstraZeneca	ChAdOx1-S ([recombinant]	Non-Replicating Viral Vector	149	73	15 February 2021	18 years and older	Six months, storage temperature: 2°C to 8°C	AstraZeneca/SK Bioscience Co. Ltd	WHO Recommendation AstraZeneca/SKBio - COVID-19 Vaccine (ChAdOx1-S [recombinant])	Oxford/AstraZeneca: Vaxzevria – COVID-19 Vaccine Tracker
Covishield	ChAdOx1-S ([recombinant]	Non-Replicating Viral Vector	49	6	15 February 2021	18 years and older	Nine months, storage temperature: 2°C to 8°C	Serum Institute of India Pvt. Ltd	COVISHIELD | WHO - Prequalification of Medical Products (IVDs, Medicines, Vaccines, and Immunization Devices, Vector Control)	Serum Institute of India: Covishield (Oxford/ AstraZeneca formulation) – COVID-19 Vaccine Tracker
Covaxin	Whole Virion Inactivated Corona Virus vaccine	Inactivated	14	16	03 November 2021	18 years and older	Nine months, storage temperature: 2°C to 8°C	Bharat Biotech International Ltd.	COVAXIN | WHO - Prequalification of Medical Products (IVDs, Medicines, Vaccines, and Immunization Devices, Vector Control)	Bharat Biotech: Covaxin – COVID-19 Vaccine Tracker
SinoPharm	COVID-19 Vaccine (Vero Cell), Inactivated	Inactivated	93	39	07 May 2021	18 years and older	24 months, storage temperature: 2°C to 8° C	Beijing Institute of Biological Products Co., Ltd. (BIBP)	COVID-19 Vaccine (Vero Cell), Inactivated	Sinopharm (Beijing): Covilo – COVID-19 Vaccine Tracker
Sinovac	COVID-19 Vaccine (Vero Cell), Inactivated	Inactivated	56	42	01 June 2021	3 to 59 years of age	24 months, storage temperature: 2°C to 8° C	Sinovac Life Sciences Co., Ltd.	CoronaVac | WHO - Prequalification of Medical Products (IVDs, Medicines, Vaccines, and Immunization Devices, Vector Control)	Sinovac: CoronaVac – COVID19 Vaccine Tracker

**Table 4:** List of coronavirus disease 2019 (COVID-19) vaccines approved worldwide

Vaccine	Approved countries	Trials	Links
Anhui Zhifei Longcom: Zifivax	4	21	https://covid19.trackvaccines.org/vaccines/27/
Bagheiat-allah University of Medical Sciences: Noora vaccine	1	3	https://covid19.trackvaccines.org/vaccines/129/
Bharat Biotech: Covaxin	14	16	https://covid19.trackvaccines.org/vaccines/9/
Bharat Biotech: iNCOVACC	1	4	https://covid19.trackvaccines.org/vaccines/87/
Biological E Limited: Corbevax	2	7	https://covid19.trackvaccines.org/vaccines/54/
CanSino: Convidecia	10	14	https://covid19.trackvaccines.org/vaccines/2/
CanSino: Convidecia Air	2	5	https://covid19.trackvaccines.org/vaccines/162/
Center for Genetic Engineering and Biotechnology: Abdala	6	5	https://covid19.trackvaccines.org/vaccines/67/
Chumakov center: KoviVac	3	5	https://covid19.trackvaccines.org/vaccines/100/
Gamaleya: Gam-COVID-Vac	1	2	https://covid19.trackvaccines.org/vaccines/191/
Gamaleya : Sputnik light	26	7	https://covid19.trackvaccines.org/vaccines/126/
Gamaleya: Sputnik V	74	25	https://covid19.trackvaccines.org/vaccines/12/
Genova Biopharmaceuticals Ltd: Gemcovac-16	1	2	https://covid19.trackvaccines.org/vaccines/200/
Health institutes of Turkey: Turkovac	1	8	https://covid19.trackvaccines.org/vaccines/77/
Instituto Finlay de Vacunas Cuba : Soberana 02	4	2	https://covid19.trackvaccines.org/vaccines/52/
Instituto Finlay de Vacunas Cuba : Soberana Plus	2	5	https://covid19.trackvaccines.org/vaccines/119/
Janssen (Johnson & Johnson)	113	26	https://covid19.trackvaccines. /vaccines/1/
Livzon Mabpharm Inc: V-01	1	7	https://covid19.trackvaccines.org/vaccines/108/
Medicago: Covifenz	1	6	https://covid19.trackvaccines.org/vaccines/26/
Medigen: MVC-COV1901	4	15	https://covid19.trackvaccines.org/vaccines/24/
Moderna: Spikevax	88	70	https://covid19.trackvaccines.org/vaccines/22/
Moderna: Spikevax Bivalent Original/Omicron BA.1	38	5	https://covid19.trackvaccines.org/vaccines/210/
Moderna: Spikevax Bivalent Original/Omicron BA.4/BA.5	33	2	https://covid19.trackvaccines.org/vaccines/224/
National Vaccine and Serum Institute: Recombinant SARS-Cov-2 Vaccine(CHO cell)	1	3	https://covid19.trackvaccines.org/vaccines/114/
Novavax: Nuvaxovid	40	22	https://covid19.trackvaccines.org/vaccines/25/
Organization of Defensive innovation and research: FAKHRAVAC (MIVAC)	1	3	https://covid19.trackvaccines.org/vaccines/97/
Oxford/AstraZeneca: Vaxzevria	149	73	https://covid19.trackvaccines.org/vaccines/4/
Pfizer/BioNtech: Comirnaty	149	100	https://covid19.trackvaccines.org/vaccines/6/
Pfizer/BioNtech: Comirnaty Bivalent Original /Omicron BA.1	35	3	https://covid19.trackvaccines.org/vaccines/223/
Pfizer/BioNtech: Comirnaty Bivalent Original /Omicron BA.4/BA.5	33	4	https://covid19.trackvaccines.org/vaccines/225/
PT Bio Farma : IndoVac	1	4	https://covid19.trackvaccines.org/vaccines/187/
Razi Vaccine and Serum Research Institute: Razi Cov Pars	1	5	https://covid19.trackvaccines.org/vaccines/82/
Research Institute for Biological Safety Problems (RIBSP): QazVac	2	3	https://covid19.trackvaccines.org/vaccines/30/
Sanofi/GSK: VidPrevtyn Beta	30	3	https://covid19.trackvaccines.org/vaccines/165/
Serum Institute of India: Covishield(Oxford/AstraZeneca formulation)	49	6	https://covid19.trackvaccines.org/vaccines/48/
Serum Institute of India: Covovax ( Novavax formulation)	6	7	https://covid19.trackvaccines.org/vaccines/123/
Shenzhen Kangtai Biological Products Co: KCONVAC	2	7	https://covid19.trackvaccines.org/vaccines/47/
Shifa Pharmed Industrial Co: COVIran Barekat	1	6	https://covid19.trackvaccines.org/vaccines/83/
Sinopharm (Beijing) Covilo	93	39	https://covid19.trackvaccines.org/vaccines/5/
Sinopharm (Wuhan): Inactivated (Vero cells)	2	9	https://covid19.trackvaccines.org/vaccines/16/
Sinovac: CoronaVac	56	42	https://covid19.trackvaccines.org/vaccines/7/
SK Bioscience Co Ltd: SKYCovione	1	7	https://covid19.trackvaccines.org/vaccines/81/
Takeda: TAK-019 (Novavax formulation)	1	3	https://covid19.trackvaccines.org/vaccines/80/
Takeda : TAK -919 (Moderna formulation)	1	2	https://covid19.trackvaccines.org/vaccines/79/
Valneva: VLA2001	33	9	https://covid19.trackvaccines.org/vaccines/69/
Vaxine/CinnaGen Co: SpikoGen	1	8	https://covid19.trackvaccines.org/vaccines/8/
Vector State Research center of Virology and biotechnology: Aurora -Cov	1	2	https://covid19.trackvaccines.org/vaccines/169/
Vector State Research center of Virology and biotechnology: EpiVacCorona	4	4	https://covid19.trackvaccines.org/vaccines/32/
WalVax: AWcorna	1	4	https://covid19.trackvaccines.org/vaccines/23/
Zydus Cadila: ZyCov-D	1	6	https://covid19.trackvaccines.org/vaccines/29/

COVOVAX

Developed by the Serum Institute of India, COVOVX received WHO EUL on December 17, 2021, approved for use in 12 years and older. This vaccine has the same formulation as the Novavax vaccine (NVX-CoV2373). It has EUL in six countries, Bangladesh, India, Indonesia, Philippines, South Africa, and Thailand. Currently has three phase-2 trials, and four phase-3 trials (https://www.ctri.nic.in/Clinicaltrials/pmaindet2.php?trialid=65971)

A phase-3, observer-blinded, randomized, active-controlled study was carried out in adults (aged ≥ 18 years) in India who had already received primary vaccination against COVID-19 at least six months ago (six months or 180 days from the second dose), to evaluate the immunogenicity and safety of the COVOVAX booster dose in comparison with the control vaccine. A total of 372 eligible participants of ≥ 18 years of age who have completed a primary 2-dose schedule of COVID-19 vaccination at least six months ago will be enrolled in this study in two cohorts of 186 participants, each with 1:1 allocation to COVOVAX or control vaccine. The primary outcome is to evaluate titers of Anti-S IgG and Neutralizing antibodies 28 days after vaccination.

Nuvaxovid (Novavax)

Also referred to as NVX-CoV2373, developed by the Serum Institute of India, it received WHO EUL recommendation on December 20, 2021 in 40 countries. There are 22 registered trials in 14 countries consisting of two phase-1, 11 phase-2, and nine phase-3 trials.

Spikevax

Developed by Moderna, it is an mRNA vaccine (nucleoside modified) approved in 88 countries. It has gone through 70 trials in 70 countries, 10 phase-1, 36 phase-2, and 24 phase-3 trials.

Comirnaty

Developed by Pfizer/BioNTech, it is an mRNA vaccine (nucleoside modified) for use in individuals aged 16 years and older. It is the first vaccine the FDA approved for emergency use in December 2020. Currently, it is approved in 149 countries with over 100 trials in 31 countries, 17 phase-1 trials, 54 phase-2 trials, and 29 phase-3 trials.

Convidecia

Developed by CanSino Biologics Inc., it is a non-replicating viral vector vaccine for use in individuals aged 18-59 years. It is approved for use in 10 countries with 14 trials in six countries, four phase-1 trials, six phase-2 trials, and four phase-3 trials.

Jcovden/Janssen

Developed by Johnson & Johnson, it is a non-replicating viral vector vaccine for use in individuals aged 18 years and older. It is approved for use in 113 countries with 26 trials in 25 countries, six phase-1 trials, 12 phase-2 trials, and eight phase-3 trials.

Vaxzevria

Developed by Oxford/AstraZeneca and Verity/SII (COVISHIELD), it is a non-replicating viral vector-based vaccine. It is approved for use in ages 18 and older. Vaxzevria has been shown to have an efficacy of 72% against symptomatic SARS-CoV-2 infection, as shown by the primary data analysis irrespective of interdose interval from trial participants who received two standard doses with an interval of 4-12 weeks. Vaccine efficacy appeared to be higher when the interval between doses was longer. It is made from a virus (ChAdOx1), a weakened version of a common cold virus (adenovirus) that causes infections in chimpanzees. It is currently approved in 149 countries. There are 73 ongoing trials in 34 countries [336,337].

Covishield

Developed by the Serum Institute of India, it is a non-replicating viral vector-based vaccine. This vaccine has the same formulation as the Oxford/AstraZeneca vaccine (AZD1222). It is also made from the ChAdOx1 virus, a weakened common cold virus (adenovirus). In addition, genetic material from the spike glycoprotein has been added to the ChAdOx1 construct. In pooled data from four trials, this vaccine had a protective efficacy of 67% (95% confidence interval [CI]: 57%-74%) for preventing symptomatic and laboratory-proven COVID-19 and nearly 100% (72-100%) for preventing hospitalizations and severe infections, beginning 21 days after the second dose. Covishield, which is identical to the Oxford-AstraZeneca (ChAdOx1 nCoV-19) vaccine in composition and immunogenicity, has accounted for nearly 88% of all doses in the country to date and has been the sole vaccine used in some areas. Covishield is approved in 49 countries. There are six ongoing trials in one country [336,337].

Covaxin

Developed by Bharat Biotech, this is an inactivated SARS-CoV-2 antigen (strain NIV-2020-770) approved for use aged 18 and older. There are currently 16 trials in two countries. Vaccine efficacy against COVID-19 of any severity, 14 or more days after the second dose, was 78%. Vaccine efficacy against severe disease is 93%. In adults aged less than 60 years, efficacy was 79%, and in those aged 60 years and over, it was 68%.

Covilo

Developed by Sinopharm (Beijing), this is an inactivated antigen of the SARS-CoV-2 WIV04 strain. A large multi-country phase-3 trial has shown that two doses, administered at an interval of 21 days, have an efficacy of 79% against symptomatic SARS-CoV-2 infection 14 or more days after the second dose. Vaccine efficacy against hospitalization was 79%. WHO recommends three to four weeks between the first and second doses of the primary series. A booster dose may be considered four to six months after the completion of the primary vaccination series, starting with the higher priority use groups, following the WHO Prioritization Roadmap. There are currently 39 trials in 18 countries [336,337].

CoronaVac

Developed by Sinovac Life Sciences Co., Ltd, this is an inactivated SARS-CoV-2 Virus (CZ02 strain). A large phase-3 trial in Brazil showed that two doses, administered at an interval of 14 days, had an efficacy of 51% against symptomatic SARS-CoV-2 infection, 100% against severe COVID-19, and 100% against hospitalization starting 14 days after receiving the second dose. The WHO Strategic Advisory Group of Experts on Immunization (SAGE) recommends the use of the Sinovac-CoronaVac vaccine in two doses (0.5 ml) given intramuscularly, an interval of two to four weeks between the first and second dose of the primary series. SAGE also recommends that a third dose of the Sinovac vaccine be offered to persons aged 60 and above as part of an extension of the primary series. Current data does not indicate the need for an additional dose in persons under 60 years of age. There are 42 trials in 10 countries, some are ongoing and others are complete [336,337].

Some of the side effects associated with COVID-19 vaccination are fever, generalized body aches, pain at the injection site, anaphylaxis, GBS, myocarditis and pericarditis, thrombosis with thrombocytopenia syndrome (TTS).

COVID-19 in special circumstances

Clinical risk factors, including diabetes, hypertension, chronic kidney disease, obesity, cancer diagnosis, and immunosuppression, have been associated with COVID-19 disease progression and death in retrospective studies. However, the risk of immunosuppression on COVID-19 disease outcomes, independent of age and comorbidities, is incompletely understood.

Immunocompromised State

Several observational studies compared the implications of COVID-19 in immunocompromised and the general population. In most of these studies, immunocompromised hosts included (i) patients undergoing active treatment for solid tumor and hematologic malignancies, (ii) recipients of solid-organ transplants, (iii) active treatment with high-dose corticosteroids, alkylating agents, antimetabolites, transplant-related immunosuppressive drugs, cancer chemotherapeutic agents classified as severely immunosuppressive, TNF blockers, and other biologic agents that are immunosuppressive or immunomodulatory, (iv) moderate or severe primary immunodeficiency (e.g., common variable immunodeficiency disease, severe combined immunodeficiency, DiGeorge syndrome, Wiskott-Aldrich syndrome), and (v) advanced or untreated HIV infection (HIV and CD4 cell counts less than 200/mm^3^) [338].

There is no evidence that immunosuppression is associated with increased mortality, especially when the well-known risk factors of age and other comorbidities are adjusted in the statistical models; however, several studies support the increased incidence of hospitalizations and prolonged recovery time.

Factors associated with higher odds of hospitalization included older age, comorbidities (hypertension/cardiovascular disease, diabetes, lung disease, renal impairment), and chronic oral glucocorticoid use (prednisone equivalent ≥10 mg/day, OR 2.05, 95%CI 1.06-3.96). The use of disease-modifying antirheumatic drugs (DMARDs), that is, no anti-cytokine biological therapies or JAK inhibitors, was associated with a reduced risk of hospitalization [339].

For the treatment of SARS-CoV-2 in people with immunocompromised states, there is no evidence to suggest that these patients should be treated any differently than the general population. Due to the longer shedding durations in immunocompromised persons, a longer course of antivirals, or initiation later in the disease, may be warranted, although these strategies are unstudied. Baseline immunosuppression may be dose-reduced, held, delayed, or replaced with COVID-19-specific therapy such as dexamethasone [340].

In summary, immunosuppressed people are not at increased risk of mortality from COVID-19 compared to age-matched and comorbidity-matched controls but are at higher risk of hospitalization from COVID-19.

Pregnancy

Pregnant women are no longer susceptible to SARS-CoV-2 compared to the general population. Over 90% of the infected pregnant women recover without being hospitalized. However, being pregnant appears to heighten the possibility of a severe clinical course of COVID-19 (increased risks of ICU admission, mechanical ventilation, and death) compared to non-pregnant women of the same age [341]. Compared to symptomatic non-pregnant women of reproductive age, pregnant women appear to be at an elevated risk of serious illness and mortality due to the possibility of rapid clinical deterioration [342,343]. Older age (≥35 years), underlying medical comorbidities (hypertension, diabetes), obesity, and being unvaccinated are risk factors for serious illness and mortality in pregnancy [344].

In a case-control study of 5183 pregnant and matched 5183 non-pregnant females, after propensity score matching for asthma, chronic obstructive lung disease, cardiovascular disease, chronic renal illness, diabetes, hypertension, obesity, smoking, immunosuppression, age, language, country, health insurance, an increased risk for death (1.5% vs. 0.8%; OR 1.84, 95%CI 1.26-2.69), pneumonia (9.9% vs. 5.6%; OR 1.86, 95%CI 1.60-2.16), intubation (8.1% vs. 8.6%; OR 0.93, 95%CI 0.70-1.25), and ICU admission (13% vs. 7.4%; OR 1.86, 95%CI 1.41-2.45) were observed [345].

In a retrospective cohort study including data from 463 hospitals in the US, maternal mortality during childbirth hospitalization prior to the pandemic rose from 5.2 deaths per 100,000 pregnant patients to 8.7 deaths per 100,000 pregnant patients during the pandemic (March 1, 2020, to April 31, 2021) (OR 1.75, 95%CI 1.19-2.58) [346].

The concern for mother-to-child transmission (vertical transmission) of COVID-19 infection is reported to be less than 2%, and the risk factors for such a transmission included severe COVID-19, admission to ICU, postnatal infection, and death (343). There is minimal coexpression of ACE-2 and TMPRSS2 in the placenta, making the entry of SARS-CoV-2, subsequent infection, and fetal transmission very infrequent [347].

The risk of developing preeclampsia is higher in women with COVID-19 infection during pregnancy, even in asymptomatic patients. A meta-analysis of 26 observational studies, including over 780,000 pregnant women with SARS-CoV-2 infection, showed a 62% higher risk of developing preeclampsia (7% vs. 4.8%; OR 1.62, 95%CI 1.45-1.82) [348].

Preterm and cesarian births were more likely in neonates delivered by mothers with symptomatic COVID-19 [344], fever, and hypoxemia implicated in the increased risk of preterm labor, premature rupture of membranes, and abnormal fetal heart rate patterns [349].

Pregnant patients with COVID-19 were at a higher risk for stillbirth in an analysis of over 1.2 million delivery hospitalizations in the US; the adjusted risk for stillbirth was higher in deliveries with COVID-19 compared with deliveries without COVID-19 during March 2020-September 2021 (adjusted relative risk (aRR) = 1.90; 95% CI = 1.69-2.15) [350]. The risk of miscarriage and congenital anomalies is not increased in pregnant patients with COVID-19.

HIV/AIDS

In the early pandemic phase, reports suggested that HIV patients had no significant differences in clinical outcomes when infected with COVID-19 compared to the general population [351,352]. However, subsequent reports suggest that HIV patients with COVID-19 are prone to worse outcomes.

Low CD4 counts or low CD4 nadirs were linked to negative outcomes (ICU admission, mechanical ventilation, and death), and this elevated risk was seen in individuals who had achieved virologic suppression of HIV [353]. It must be noted that lymphopenia is often seen in patients with COVID-19 infection; hence, obtaining CD4 counts may not reflect a patient’s HIV disease stage accurately.

After controlling for demographics, smoking, and the presence of comorbidities, database research of more than one million COVID-19 cases in the US found that COVID-19-associated hospitalization and death were greater in patients with HIV than in those without HIV [354].

COVID-19 management in patients with HIV does not differ from the general population. Close monitoring of potential drug-drug interactions between antiretroviral treatments (ART) and COVID-19 treatments must be maintained. Dexamethasone use should be done in consultation with HIV specialists and restricted to only one dose in HIV patients on rilpivirine as part of the ART, as it could lower the levels of rilpivirine.

HIV patients on ART must be encouraged to continue taking them, as treatment interruption may lead to rebound viremia. New HIV patients, or those not on ART, with COVID-19 must be started on ART after a discussion with an HIV specialist since the timing of initiation of ART in these patients remains unclear [355].

COVID-19 and mental health

It is well known from prior studies that natural disasters and economic crises result in an increased diagnosis of anxiety, depression, PTSD, substance abuse, and suicidal tendencies [356,357]. During the COVID-19 pandemic, the mandatory isolation following the diagnosis, prolonged hospitalization, the ensuing stigma and discrimination, and a lack of social support influence mental health in this population [358]. In a study involving over 2000 healthcare workers compared to over 250 non-clinical staff, there were notable differences in the intensity of dread, anxiety, and depression. Additionally, front-line medical staff with frequent contact with infectious patients, such as those working in the ED, infectious disease, and ICU, demonstrated higher scores on the fear Hamilton Anxiety Scale (HAM-A) and the Hamilton Depression Scale (HAM-D) and they were 1.4 times more likely to experience fear and twice as likely to experience anxiety and depression compared to non-clinical staff [359].

Patients diagnosed with COVID-19 and those who survive the infection are at an increased risk of psychiatric symptoms and disorders. In a retrospective study analyzing administrative healthcare data, the investigators looked at the risk of incident mental illnesses in patients (n > 150,000) who survived COVID-19 for 30 days and patients (n > 5,600,000) without COVID-19 [360]. After adjusting for potential confounding factors (e.g., age, smoking status, and general medical comorbidities), there was an increased risk of a psychiatric diagnosis in patients surviving COVID-19 (HR 1.46, 95%CI 1.40-1.52). The most common issues encountered in these patients were cognitive dysfunction, anxiety, depression, PTSD, sleep disorders, and substance abuse.

Since the epidemic, there has been a rise in microaggressions, hate crimes, verbal attacks, and acts of physical violence against Asian Americans [361]. This fact must be acknowledged. Exclusionary policies, disparaging rhetoric, and implicit encouragement at federal and institutional levels, which act to perpetuate anti-Asian violence and xenophobia, have resulted in the stigmatization of persons of Asian origin as a result of the global health crisis [362].

Long-term effects, complications, and mortality

Nearly three years after the pandemic's start, there has been new information about the long-term complications of COVID-19, especially Long COVID. A proportion of patients have been noted to have a similar symptom profile that persists long after they have recovered from the infection without returning to their baseline health. Negative virus isolation tests confirm recovery. According to the CDC, post-acute COVID-19 is the term used to describe these symptoms, which last for more than four weeks after the initial infection. It includes Long COVID, multiorgan effects, and effects of treatment. The effects of treatment are similar to the sequela of any severe infection, including deconditioning, PTSD, and weakness. Long COVID can be further divided based on the time duration as subacute (up to 12 weeks from the infection) and chronic (when symptoms persist more than 12 weeks) [363,364].

Long COVID-19 is known to affect all major organ systems, not limited to cardiorespiratory but including neurological, renal, musculoskeletal, endocrine, and gastrointestinal systems. It is a constellation of symptoms, mainly fatigue (seen in more than 50% of patients) and shortness of breath. Other symptoms include arthralgias, cognitive delay, mental health disorders, altered smell and taste, chronic dry cough, sleep disturbances, and hair loss. It also affects survivors irrespective of their disease severity. This syndrome of fatigue post-infection is very common with various viruses such as MERS, SARS, influenza, Ebola virus, and EBV, to name a few [365]. Long COVID affects people of all age groups, although some studies noted a correlation between older age, female sex, severity of symptoms, need for oxygen, and comorbidities, especially respiratory conditions [366,367]. COVID-19 is known to have a milder effect on children. Studies show that children (<18 years) seem to suffer from Long COVID rarely, and the few children who do get it recover sooner from the symptoms in around one to five months [368].

The exact pathophysiology of this process is still under evaluation, but numerous theories have been proposed in this regard. One theory believes in the persistence of the virus in the tissues, which could cause long-term stimulation of antigenic receptors and continued symptoms with a negative COVID-19 test swabbed from the nasopharynx. Several autopsies have shown the virus to persist in respiratory tissues, kidneys, and intestines [369,370]. This prolonged immune system stimulation gradually leads to immune dysregulation, immune exhaustion, and autoimmunity. Patients affected by severe COVID-19 are noted to have a hyperinflammatory cytokine response. This strongly supports the theory that chronic inflammation is ongoing even after recovery from the active infection, which causes the symptoms of fatigue and arthralgias. COVID-19 also decreases the ACE-2 receptor expression, which causes an imbalance in the renin-angiotensin system, which alters the physiology [369]. A study also noted that post-COVID-19 patients with symptoms had a lower nitrate level and a higher nitrite level than the uninfected group, which could suggest the use of nitrates as a predictor for health status follow-up [371].

Reactivation of the virus is another concern in patients with Long COVID. It would be difficult to prove the origin of the virus reactivation versus infection with a new strain. However, it does bring up the possibility of carriers. Given the symptom severity of Long COVID and other unknown possibilities, patients with Long COVID should be closely monitored [372]. Long COVID complications are given in Table 5.

**Table 5:** Long-COVID complications COVID: coronavirus disease

System	Long-COVID Complications
Neuropsychiatric [365,373-377]	Chronic fatigue syndrome, Headache, Myelitis, Neuropathies, Paresthesia, Parkinsonism, Cogwheel rigidity, Optic neuritis, Anosmia, Ageusia, Encephalitis, Epilepsy, Bell’s palsy, Myoclonus, Transient ischemic attack, Stroke, Depression, Anxiety.
Pulmonary [153,378-381]	Pulmonary fibrosis, Pulmonary thromboembolism, Acute respiratory distress syndrome, Pulmonary embolism, Pneumothorax
Cardiovascular [12,13]	Dysrhythmias, Atrial fibrillation/flutter, Ventricular arrhythmias, Sinus tachycardia/bradycardia, Inflammatory heart disease, Pericarditis/myocarditis, Coronary artery disease, Cardiomyopathy (ischemic/nonischemic)
Endocrine [14]	Fulminant type diabetes, autoimmune diabetes, new-onset transient hyperglycemia
Rheumatology [382-384]	Polyarthritis, Osteoporosis, Osteonecrosis

Myths associated with COVID-19

There has been much mystery surrounding COVID-19 infection, especially the virus's origin, leading to many myths. We have attempted to demystify some of the common myths associated with COVID-19.

COVID-19 Virus was Synthesized by Laboratory Manipulation of a Related Coronavirus

Genetic research showed evidence that COVID-19 is not derived from any known virus, so the possibility of laboratory manipulation would be doubtful. WHO experts also support this information via a special commission investigation report. The existing theory about the virus's origin is that it is an animal virus that got transmitted to humans, a process known as zoonosis [385].

Ibuprofen Should be Avoided in COVID-19 Since it Reacts with the Virus

The role of non-steroidal anti-inflammatory drugs (NSAIDs) such as ibuprofen in the immune response to COVID-19 is unclear. However, it does reduce the production of inflammatory cytokines resulting in the complications associated with COVID-19, such as ARDS. Also, the ACE-2 receptor is crucial in the pathogenesis of respiratory symptoms, and the use of ibuprofen does not appear to increase the expression of ACE-2 receptors. Hence, ibuprofen can be safely used to manage COVID-19 symptoms [386].

The Virus is Only 100 nm (0.1 mm) in Size, so Filters and Masks will not Work

SARS-CoV-2 is transmitted in the form of respiratory droplets approximately 0.3-0.5 mm in size. A droplet contains respiratory secretions, including water, protein, and salt. There is enough evidence to prove that masks can prevent virus spread from asymptomatic carriers. Along with masks, social distancing and hand hygiene help prevent virus transmission [387,388]. It is also a myth that wearing a face mask would increase the level of CO2 in the blood [389].

Sanitizer Solutions Should be Used to Disinfect Hands

Using soap and water to wash hands is as effective as cleaning hands with an alcohol-based sanitizer. Hand hygiene, along with social distancing, helps reduce virus transmission. Using alcohol and chlorine-based sprays in high concentrations for disinfection can be harmful if used on clothes or mucous membranes [388,389].

A Positive PCR Test is Equivalent to an Active Infection

Patients with a positive PCR test with no active symptoms of COVID-19 can either be carriers or in a convalescent phase after a recent infection. Reinfection would be a possibility, but it is very unlikely. A study on patients discharged from the hospital after COVID-19 infection noted virus presence in the sputum and faeces for a few weeks after discharge [390].

Patients Without Symptoms Cannot Spread the Infection

Most asymptomatic patients and those with mild symptoms can still spread the disease. Hence, if infection is suspected, there is a need to follow precautions such as hand hygiene and adequate social distancing [388].

The Use of Vitamins and Minerals Can Prevent COVID-19 Infection

Vitamins such as C and D, zinc, elderberry supplements, colloidal silver, and more have been thought to be beneficial for COVID-19 prevention and management of infection. CITRIS ALI trial showed no evidence of administering 50 mg/kg vitamin C for four days to alter the disease course or severity of acute respiratory failure [391]. Vitamin D, if low, can cause acute respiratory infections with the risk of respiratory distress, but there is no evidence of benefit in COVID-19 infection. Zinc inhibits virus multiplication and has a role in treating the common cold. FDA declared colloidal silver as unsafe, although it is an antibacterial and an antiviral agent. Elderberry has antiviral and immunomodulatory properties, a daily dose of 600 mg for 10 days can reduce the duration of cold symptoms. It needs to be cooked, and improper cooking has risks of causing immunosuppression [392].

Hydroxychloroquine Can Prevent COVID-19 Infection

The COVID-19 pandemic led to a rapid search for effective treatment options, and drug repurposing appeared to be the fastest solution. Anti-malarial drugs such as chloroquine and hydroxychloroquine were noted to inhibit COVID-19 replication in vitro. Hydroxychloroquine was found to be more potent than chloroquine. The studies had major limitations, with short treatment periods and a high risk of bias. There is no definite proof to prove that hydroxychloroquine can be used for chemoprophylaxis [393,394].

Social Distancing of 6 Feet is Always Protective

A physical distance of at least 6 feet between two people is considered safe since it is assumed that respiratory droplets would not be able to travel the distance and infect other people. Recent studies show the ability of COVID-19 to aerosolize, meaning they can travel longer than 6 feet, but this is not the primary mode of infection transmission [395].

## Conclusions

COVID-19 emerged as a multifaceted disease caused by the novel coronavirus, SARS-CoV-2. Its impact was profound, characterized by substantial mortality rates and severe economic repercussions worldwide. Extensive literature exists, encompassing a wide spectrum of aspects related to COVID-19, ranging from its origins and pathogenesis to organ-specific manifestations, treatment options, and vaccination strategies. This comprehensive review aims to synthesize the available literature, offering a detailed account of the infection, including its roots, progression, modes of transmission, epidemiological trends, organ involvement, therapeutic interventions such as vaccines, and its occurrence in unique circumstances.

Over the course of three years, despite remarkable progress in diagnostic methods, targeted therapeutic advancements remain in the research phase. Nevertheless, the development and widespread distribution of highly effective vaccines significantly bolstered immunity among populations, alleviating the strain on healthcare systems worldwide. While the WHO has declared that COVID-19 is no longer a global health emergency, it underscores the ongoing vigilance required due to the emergence of new variants that can lead to fresh outbreaks and casualties.
